# EZH2 inhibition suppresses endometrial cancer progression via miR-361/Twist axis

**DOI:** 10.18632/oncotarget.14586

**Published:** 2017-01-10

**Authors:** Kei Ihira, Peixin Dong, Ying Xiong, Hidemichi Watari, Yosuke Konno, Sharon JB Hanley, Masayuki Noguchi, Noriyuki Hirata, Futoshi Suizu, Takahiro Yamada, Masataka Kudo, Noriaki Sakuragi

**Affiliations:** ^1^ Department of Gynecology, Hokkaido University School of Medicine, Hokkaido University, Sapporo 0608638, Japan; ^2^ Department of Women's Health Educational System, Hokkaido University School of Medicine, Hokkaido University, Sapporo 0608638, Japan; ^3^ Department of Gynecology, State Key Laboratory of Oncology in South China, Sun Yat-Sen University Cancer Center, Guangzhou 510060, P. R. China; ^4^ Division of Cancer Biology, Institute for Genetic Medicine Hokkaido University N15, W7, Sapporo 0608638, Japan

**Keywords:** EZH2, GSK343, miR-361, endometrial cancer, 5-AZA-CdR

## Abstract

EZH2 inhibition and reactivation of tumor suppressor microRNAs (miRNAs) represent attractive anti-cancer therapeutic strategies. We found that EZH2-suppressed let 7b and miR-361, two likely tumor suppressors, inhibited endometrial cancer (EC) cell proliferation and invasion, and abrogated cancer stem cell-like properties. In EC cells, EZH2 induced and functioned together with YY1 to epigenetically suppress miR-361, which upregulated Twist, a direct target of miR-361. Treating EC cells with GSK343, a specific EZH2 inhibitor, mimicked the effects of siRNA-mediated EZH2 knockdown, upregulating miR-361 and downregulating Twist expression. Combining GSK343 with 5 AZA-2′-deoxycytidine synergistically suppressed cell proliferation and invasion *in vitro*, and decreased tumor size and weight in EC cell xenografted mice. Quantitative real-time PCR analysis of 24 primary EC tissues showed that lower let-7b and miR-361 levels were associated with worse patient outcomes. These results were validated in a larger EC patient dataset from The Cancer Genome Atlas. Our findings suggest that EZH2 drives EC progression by regulating miR-361/Twist signaling, and support EZH2 inhibition as a promising anti-EC therapeutic strategy.

## INTRODUCTION

Endometrial cancer (EC) is a heterogeneous disease characterized by dysregulated cell proliferation and metastasis [1]. While alterations in molecular pathways, such as proto-oncogene activation, tumor suppressor gene inactivation, aberrant DNA methylation, and non-coding microRNA (miRNA) dysregulation, can initiate and promote EC [1], the genetic and epigenetic basis of EC is not yet fully understood. Epithelial-mesenchymal transition (EMT) is a critical early step in cancer metastasis. We have demonstrated that miR-106b, -130b, and -194 serve as key tumor suppressors by directly targeting EMT inducers, such as TWIST1 (Twist), ZEB1 and BMI-1, in EC cells [2–4].

Enhancer of zeste homolog 2 (EZH2), a crucial component of polycomb repressive complex 2, is highly expressed in multiple cancer types, making it an attractive therapeutic target in tumor treatment [5]. We report that miR-101 can suppress EC cell proliferation, invasion and stem cell-like features by targeting the oncogene *EZH2*, and downregulating Twist expression [6]. EZH2 mainly acts as an epigenetic suppressor, repressing tumor suppressor gene expression by catalyzing histone H3 methylation at lysine 27 (H3K27me3) [5]. EZH2 silences several miRNAs in various human cancers [7], suggesting that it may indirectly activate important oncogenes through modulating miRNAs. Thus, we investigated whether EZH2 activates downstream oncogenic networks to promote EC progression by downregulating potential tumor suppressor miRNAs.

We identified tumor-suppressive let-7b and miR-361 as EZH2-downregulated miRNAs that attenuate EC cell proliferation, invasiveness, and cancer stem cell-like properties. We further demonstrated that EZH2 upregulates expression of Twist, a direct target of miR-361, via direct repression of miR-361 through a YY1-dependent mechanism. EZH2 inhibition by the specific inhibitor GSK343 is sufficient to induce miR-361 expression, decrease Twist levels and inhibit EC cell proliferation and invasiveness *in vitro*. We report that EZH2 directly downregulates miR-361, a novel Twist suppressor, in EC cells. Our results support the potential clinical use of GSK343 for targeting EZH2 in EC and other human cancers.

## RESULTS

### let-7b and miR-361 are tumor suppressors potentially regulated by EZH2

**Figure 1:**
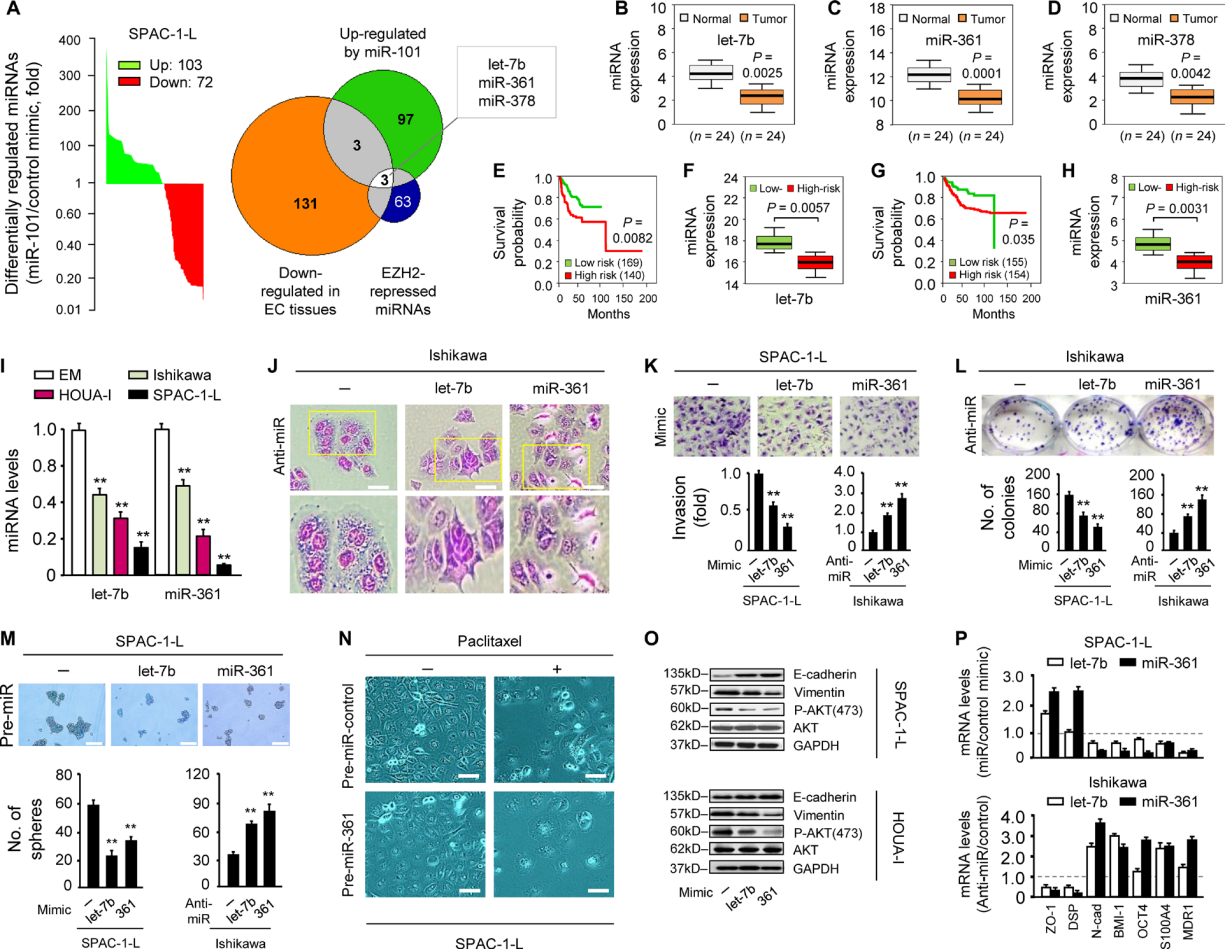
let-7b and miR-361 are tumor suppressors potentially regulated by EZH2 (**A**) miRNA profiling showing miRNAs (103 upregulated and 72 downregulated) differentially expressed in miR-101 mimic-transfected SPAC-1-L cells compared with controls Venn diagram depicts overlapping miRNAs (let-7b, miR-361 and miR-378) that may be EZH2-regulated tumor suppressors. Expression of let-7b (**B**), miR-361 (**C**) and miR-378 (**D**) as assessed by qRT-PCR in 24 EC and adjacent normal tissues. EC patient survival curves based on poor outcome risk from TCGA data were created using the SurvMicro database. Box plots show lower let-7b (**E**–**F**) or miR-361 (**G**–**H**) levels in high-risk patients. (**I**) qRT-PCR analysis of let-7b and miR-361 in three EC cell lines and EM cells. (**J**) Morphology of Ishikawa cells transfected with synthetic let-7b, miR-361 inhibitors (anti-miRNA), or negative control miRNA inhibitor. Scale bars represent 30 μm. (**K**) Relative invasion of SPAC-1-L or Ishikawa cells after overexpression or knockdown of let-7b or miR-361, respectively. (**L**) Clone formation assays with SPAC-1-L cells overexpressing let-7b or miR-361, and let-7b or miR-361 knockdown Ishikawa cells. (**M**) Sphere formation assays after transfection of SPAC-1-L and Ishikawa cells with either miRNA mimics or anti-miRNA inhibitors. Scale bars represent 100 μm. (**N**) miR-361 sensitized SPAC-1-L cells to paclitaxel Scale bars represent 30 μm. (**O)** Immunoblot of indicated proteins in EC cell lines after transduction with let-7b, miR-361 or control mimic (**P**) qPCR analysis of indicated mRNAs in SPAC-1-L cells after let-7b or miR-361 overexpression, or in let-7b or miR-361 knockdown Ishikawa cells. All gene expression changes shown here were significant. ^*^P < 0.01.

We showed that miR-101 directly targets EZH2 [6], which controls expression of a host of miRNAs [7], indicating possible overlap between miRNAs upregulated by miR-101 and those induced upon EZH2 knockdown. To assess global changes in miRNA levels following transient miR-101 mimic overexpression, microarray-based miRNA profiling of the EC cell line, SPAC-1-L, was performed to generate a list of 175 miRNAs. miR-101 restoration upregulated 103 miRNAs and downregulated 72 (Figure 1A; Supplementary Table 1). We then used an integrative approach to search for potential tumor suppressor miRNAs regulated by EZH2. The group of 103 miRNAs overlapped with miRNAs exhibiting lower endogenous levels in EC relative to normal tissues [8], leading to the identification of six miRNAs. Of these, three (let-7, miR-361 and miR-378) were upregulated by EZH2 knockdown in DU145 prostate cancer cells [7], and were selected as the top candidates for further investigation (Figure 1A).

Using a quantitative real-time PCR (qRT-PCR) assay, we validated that let-7b, miR-361, and miR-378 levels in 24 primary ECs were reduced compared to adjacent normal tissues (Figure 1B–1D). We evaluated the clinical significance of this finding through analysis of The Cancer Genome Atlas (TCGA) dataset in 309 EC samples. Lower let-7b (Figure 1E–1F) and miR-361 (Figure 1G–1H), but not miR-378 levels, were associated with worse outcomes (high-risk group). Similar analysis of TCGA datasets comprising multiple cancer types also demonstrated reduced let-7b and miR-361 expression in high-risk cancers (Supplementary Figure 1).

We first evaluated let-7b and miR-361 expression in three EC cell lines and the immortalized human endometrial epithelial cell line, EM, using qRT-PCR. Both let-7b and miR-361 levels were lower in EC than in EM cells (Figure 1I), indicating that these two miRNAs may function as tumor suppressors. To elucidate their biological functions, we transiently knocked them down with anti-miRNA inhibitors in less-aggressive Ishikawa cells, which have high endogenous let-7b and miR-361 levels (Supplementary Figure 2A, left panel). let-7b or miR-361 downregulation led to a more scattered and mesenchymal morphology, a hallmark of the EMT process (Figure 1J), and promoted Ishikawa cell invasion and proliferation (Figure 1K–1L). In contrast, in aggressive SPAC-1-L cells expressing the lowest let-7b and miR-361 levels, transient overexpression of either miRNA using mimics suppressed *in vitro* cell invasion and proliferation (Figure 1K–1L and Supplementary Figure 2A, right panel). We further tested whether let-7b and miR-361 inhibited cancer stem-like phenotypes and drug resistance. A sphere formation assay revealed that let-7b or miR-361 overexpression suppressed SPAC-1-L sphere formation and sensitized cells to paclitaxel. We also observed increased sphere-forming capacity and reduced sensitivity to paclitaxel in let-7b or miR-361-inhibited Ishikawa cells (Figure 1M–1N and Supplementary Figure 2B).

We then tested the effects of let-7b and miR-361 on EMT markers and PI3K/AKT signaling. let-7b and miR-361 overexpression in SPAC-1-L and HOUA-I cells enhanced E-cadherin expression and downregulated Vimentin and phospho-AKT (Figure 1O). In the presence of let-7b or miR-361, epithelial marker (*E-cadherin*, *ZO-1* and *DSP*) mRNAs were upregulated, whereas mesenchymal, stemness, and drug resistance markers (*Snail*, *N-cadherin*, *BMI1*, *S100A4*, *OCT4* and *MDR1*) were downregulated in SPAC-1-L cells (Figure 1P and Supplementary Figure 2C). These changes were reversed by anti-let-7b and anti-miR-361 expression in Ishikawa cells (Figure 1P and Supplementary Figure 2C). This supports the idea that let-7b and miR-361 function as tumor suppressors, maintaining the epithelial phenotype and inhibiting PI3K/AKT signaling in EC. EZH2 overexpression resulting from miR-101 loss could indirectly activate important oncogenes via suppression of let-7b or miR-361.

### miR-361 directly targets Twist and modulates downstream genes

**Figure 2:**
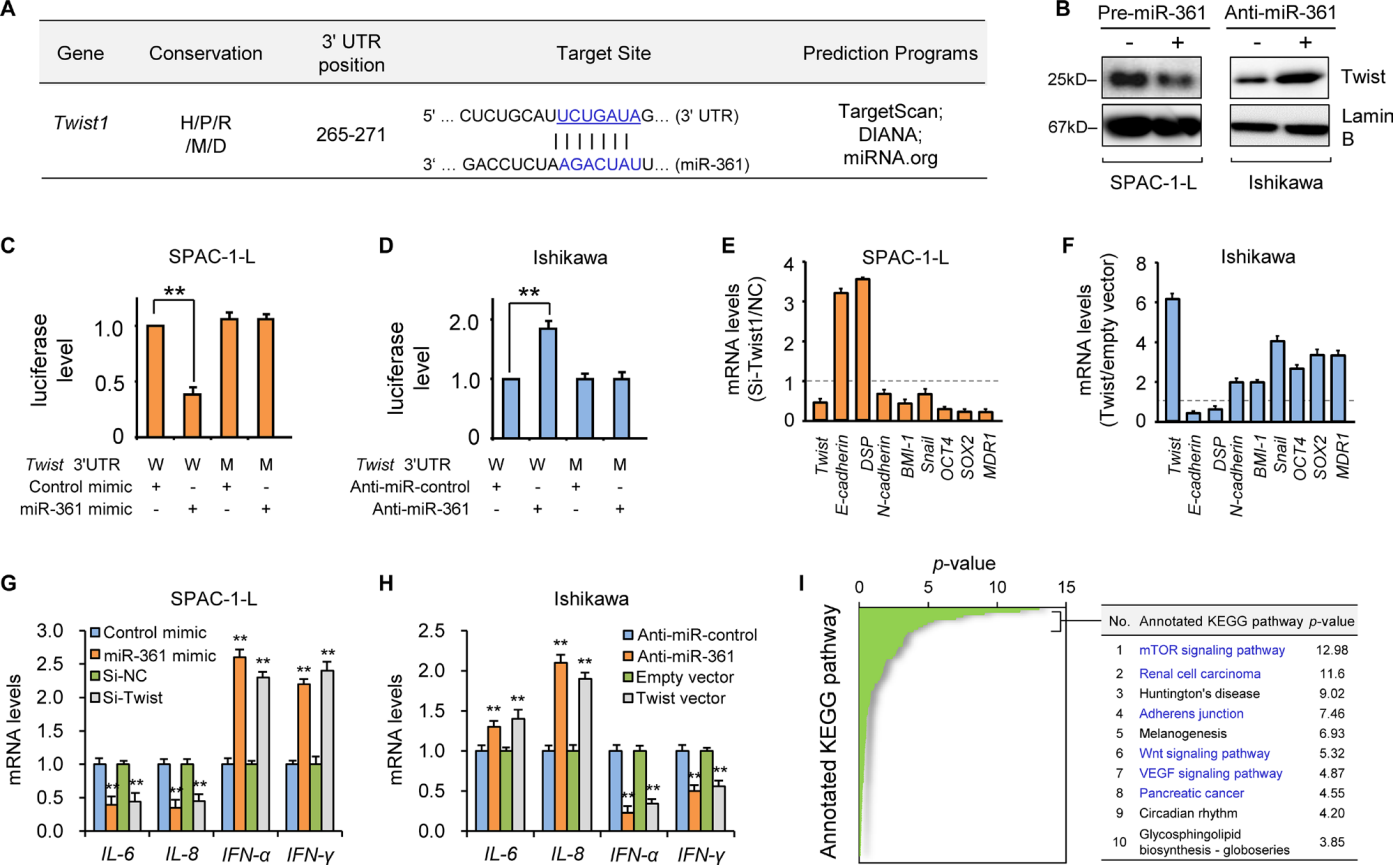
MiR-361 directly targets Twist and modulates its downstream genes (**A**) Schematic representation of putative miR-361 target site within the human *Twist* 3′-UTR as predicted by three computational databases. The miR-361 seed sequence is evolutionarily conserved between human (H), pig (P), rat (R), mouse (M), and dog (D). (**B**) Western blotting analysis of Twist in SPAC-1-L or Ishikawa cells after miR-361 overexpression or knockdown. Reporter constructs containing either wild-type (W) *Twist* 3′-UTR, or with mutation (M) at the predicted miR-361 target sequence were co-transfected into SPAC-1-L (**C**) or Ishikawa (**D**) cells, along with miR-361 mimic, anti-miR-361 inhibitor, or negative control. Relative luciferase activity was assayed. qRT-PCR analysis of EMT, invasion and stemness-related genes (normalized to *GAPDH*) in SPAC-1-L cells (**E**) upon Twist knockdown, or in Ishikawa (**F**) cells overexpressing Twist. SPAC-1-L (**G**) and Ishikawa (**H**) cells were transfected as indicated, and inflammatory genes (IL-6/8 and IFN-α/γ) were measured via qRT-PCR. (**I**) *In silico* prediction and molecular pathway enrichment analysis was performed on miR-361 predicted target genes. The top 10 ranking KEGG pathways are listed. ^*^P < 0.01.

Given that let-7b is a known tumor suppressor [9], we focused on miR-361 to determine its molecular targets. We used computational target prediction algorithms to identify candidate targets. 200 targets shared by all algorithms included the reported miR-361 target, VEGFA [10] (Supplementary Figure 3A). VEGFA inhibition by miR-361 was validated in two of three EC cell lines examined (Supplementary Figure 3B). *Twist*, a novel oncogene that promotes EC cell EMT and invasion [2], was predicted to be a direct miR-361 target (Figure 2A and Supplementary Figure 3A). Ectopic expression of the miR-361 precursor reduced Twist mRNA and protein levels in SPAC-1-L cells, while miR-361 inhibition upregulated Twist in Ishikawa cells (Figure 2B and Supplementary Figure 3C). To investigate whether *Twist* is a direct target of miR-361, a luciferase reporter vector containing the *Twist* 3′-untranslated region (UTR) and miR-361 mimic were co-transfected into SPAC-1-L cells. miR-361 suppressed *Twist* 3′-UTR reporter activity (Figure 2C). Mutating the miR-361-binding site in the *Twist* 3′-UTR eliminated luciferase repression by miR-361 (Figure 2C). miR-361 inhibition in Ishikawa cells increased *Twist* 3′-UTR luciferase activity. Mutation of the miR-361 seed sequence prevented miR-361-dependent regulation of luciferase activity (Figure 2D). This confirmed the direct repression of *Twist* by miR-361.

We previously showed that Twist promotes endometrioid EC cell EMT and invasion [2]. Here, we examined whether Twist downregulation is responsible for miR-361-mediated tumor suppression in aggressive, serous EC SPAC-1-L cells. siRNA-mediated Twist knockdown reduced cell invasion, migration, and sphere formation similarly to miR-361 overexpression, with consequent effects on Twist-regulated downstream genes (Figure 2E and Supplementary Figure 4A–4E). In contrast, ectopic Twist expression mimicked the effects of miR-361 inhibition on downstream gene expression in Ishikawa cells (Figure 2F, and Supplementary Figure 4A and 4F). Together, these data suggested that miR-361 overexpression impairs EMT in EC cells by directly targeting Twist and indirectly up-regulating epithelial markers, such as E-cadherin.

Although Twist-induced EMT and stemness could account for the malignant phenotypes caused by miR-361 loss, miR-361 may also limit EC progression through other mechanisms. Tumor cells undergoing EMT can remodel their microenvironment via enhanced secretion of multiple angiogenesis- and metastasis-promoting cytokines, chemokines and angiogenic factors [11]. Twist modulates expression of many microenvironmental genes involved in angiogenesis, local inflammatory response, and immunosuppression, such as VEGFA [12], IL-8 [13] and IFN-γ [14].Similar to miR-361 overexpression, Twist knockdown in SPAC-1-L cells downregulated pro-angiogenic and immunosuppressive cytokines (*IL-6* and *IL-8*), and upregulated *IFN-*α and *IFN-*γ, which are known to stimulate antitumor immunity and process anti-angiogenic effects [15] (Figure 2G and Supplementary Figure 5). Twist overexpression in Ishikawa cells phenocopied the effects of anti-miR-361 inhibitor treatment and induced *IL-6* and *IL-8* expression, but reduced *INF-*α and *IFN-*γ (Figure 2H and Supplementary Figure 5). These data indicate that miR-361 mediates aspects of the tumor microenvironment through Twist regulation. We also analyzed KEGG pathway enrichment using DIANA-mirPath to explore predicted miR-361 target gene biological pathways. These genes were most significantly enriched in well-known oncogenic pathways associated with tumor growth, survival, and metastasis, including the mTOR, renal cell carcinoma, adherens junction, Wnt, VEGF, and pancreatic cancer pathways. These data collectively suggest that miR-361 attenuates EC cell invasion and metastasis through modulation of Twist-dependent and -independent pathways, and potentially reprograms the tumor microenvironment, resulting in tumor regression.

### EZH2 binds to and transrepresses the miR-361 promoter via YY1

**Figure 3:**
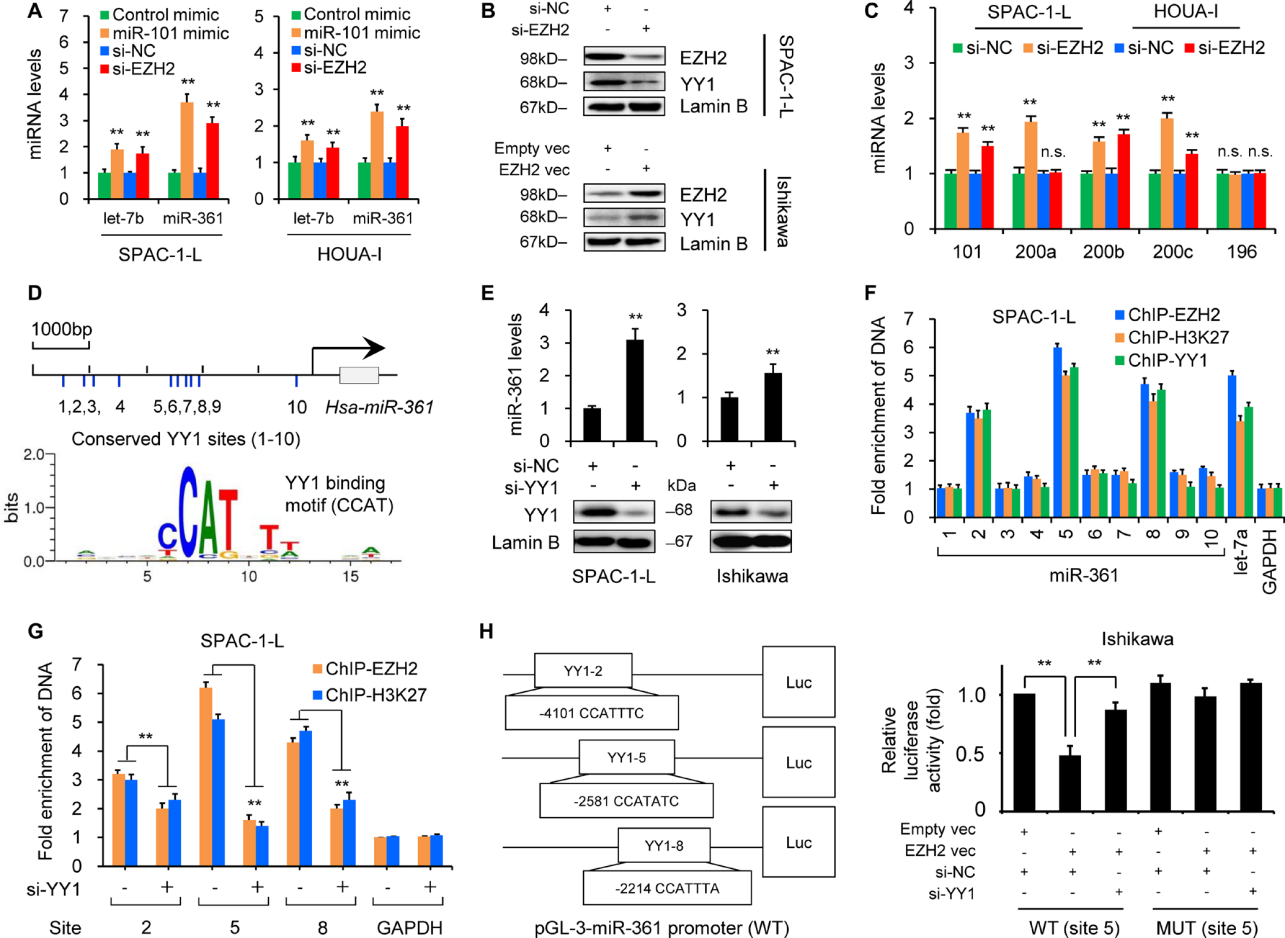
EZH2 epigenetically silences miR-361 via YY1 (**A**) qRT-PCR results showing miR-361 induction in SPAC-1-L and HOUA-I cells transfected with miR-101 mimic, EZH2 siRNA (Si-EZH2) in compared to controls (control mimic or control siRNA (Si-NC). (**B**) EZH2 and YY1 expression in SPAC-1-L or Ishikawa cells after EZH2 knockdown or overexpression as measured by western blotting. (**C**) qRT-PCR analysis in SPAC-1-L and HOUA-I cells transfected with Si-EZH2 or Si-NC. Note that miR-196 was not affected by Si-EZH2 treatment. (**D**) Schematic showing predicted YY1 binding sites (1–10) upstream of *miR-361*. TRANSFAC search identified the binding motif (CCATNTW) involved in YY1-mediated suppression. (**E**) miR-361 levels in SPAC-1-L and Ishikawa cells increased following YY1 siRNA (Si-YY1) transfection. (**F**) Chromatin from SPAC-1-L cells was immunoprecipitated with antibodies against EZH2, H3K27me3 or YY1. Purified DNA was analyzed by real-time PCR using primers amplifying regions across the *miR-361* locus. Results are expressed as fold enrichment over IgG control and are normalized to *GAPDH* promoter (negative control). Known YY1 target gene, *let-7a*, was the positive control. (**G**) SPAC-1-L cells were treated with Si-YY1 or Si-NC, and ChIP assays were conducted using EZH2 or H3K27me3 antibodies to detect EZH2 or H3K27me3 binding to *miR-361*. (**H**) Pri-miR-361 promoter luciferase reporter vectors containing YY1 binding site (2, 5 or 8) were constructed. Ishikawa cells were transfected with wild type (WT) or mutant (MUT) pri-miR-361 promoter luciferase reporter vector (site 5) along with EZH2 vector, empty vector, Si-YY1, or Si-NC. Relative luciferase activities are shown compared to empty vector and Si-NC transfection, where luciferase activity values were set to 1. ^*^P < 0.01.

Our microarray and qRT-PCR analyses demonstrated that the miR-101 mimic, a direct regulator of EZH2 [6], upregulated let-7b and miR-361 (Figures 1A and 3A). Given that EZH2 epigenetically suppresses multiple tumor suppressor miRNAs [7], we assessed whether EZH2 silenced let-7b and miR-361 in EC cells. Both let-7b and miR-361 were upregulated following EZH2 knockdown (Figure 3A–3B and Supplementary Figure 6A). miR-101 and miR-200a/b, known EZH2-suppressed miRNAs [7, 16], were used as positive controls (Figure 3C).

To investigate the molecular mechanism by which EZH2 downregulates miR-361, we retrieved the promoter sequence (5000 bp) upstream of *miR-361* and searched for all potential transcriptional factor binding sites using the TRANSFAC database. We found 10 binding sites (CCAT) for YY1 (Figure 3D and Supplementary Figure 6B), which recruits the polycomb complex to repress let-7a and miR-29b/c [17, 18], and hypothesized that YY1 may play a role in EZH2 recruitment to the *miR-361* promoter. qRT-PCR results demonstrated miR-361 upregulation after siRNA-mediated YY1 knockdown (Figure 3E), suggesting that YY1 might regulate miR-361 in EC cells. To determine whether EZH2 and YY1 associate with the *miR-361* promoter *in vitro*, we performed chromatin immunoprecipitation (ChIP)-qPCR assays with antibodies against EZH2, H3K27me3, and YY1 using lysates from SPAC-1-L cells that express endogenous EZH2 and YY1. EZH2, H3K27me3, and YY1 occupied the region of the *miR-361* promoter (sites 2, 5 and 8) similar to EZH2/H3K27me3/YY1 binding to the *let-7a* promoter [17] (Figure 3F). YY1 knockdown significantly reduced EZH2 and H3K27me3 recruitment to the *miR-361* promoter (site 2, 5 and 8; Figure 3G). Because EZH2 induces MYC expression and interacts with MYC to form a co-repressor complex that downregulates miR-29 [19], we examined whether EZH2 acts as an upstream modulator of YY1. Western blotting showed that YY1 expression decreased upon EZH2 knockdown in SPAC-1-L cells, and increased following transient EZH2 overexpression in Ishikawa cells (Figure 3B), suggesting that EZH2 induces and functions together with its recruiter, YY1, to silence miR-361.

To test whether EZH2 directly represses *miR-361* transcription, we cloned the three binding sites (2, 5 and 8) into a pGL3 luciferase vector (Figure 3H) and used site-directed mutagenesis to generate mutations targeting YY1 binding sites. Either wild type or mutant miR-361-promoter plasmids were co-transfected into Ishikawa cells along with an EZH2 expression vector and YY1 siRNA. Wild type promoter reporter activities were suppressed by EZH2 overexpression, and YY1 knockdown eliminated EZH2-induced transcriptional repression. However, mutated-type promoter luciferase activity was not affected by EZH2 overexpression or YY1 inhibition (Figure 3H and Supplementary Figure 7A), suggesting that EZH2 silences miR-361 transcription in a YY1-dependent fashion. In line with the oncogenic role of YY1 in EC [20], we verified that YY1 depletion effectively attenuated EC cell proliferation and invasion (Supplementary Figure 7B–7C). Reactivating miR-361 by targeting its upstream regulator, EZH2, may have promising therapeutic potential against EZH2-active or -overexpressing tumors.

### EZH2-induced invasion and stemness require miR-361 inhibition

To determine whether EZH2-induced malignancy requires miR-361 silencing, we performed cell invasion and sphere formation assays using SPAC-1-L cells transfected with EZH2 or control siRNA, with or without anti-miR-361 inhibitor.EZH2 inhibition reduced self-renewal and Twist expression, which was partially reversed by miR-361 knockdown (Supplementary Figure 8A and 8C). Moreover, EZH2 overexpression promoted Ishikawa cell invasion and increased Twist expression; however, miR-361 induction abrogated these effects (Supplementary Figure 8B and 8D). We then examined whether EZH2 regulates miR-361 downstream targets. EZH2 silencing in SPAC-1-L cells upregulated *E-cadherin* and downregulated mesenchymal/stem cell markers and *MMSET* (a target of EZH2), similar to the effects of miR-361 mimics. The opposite effect was detected in Ishikawa cells overexpressing EZH2 (Supplementary Figure 8E). These data demonstrated that miR-361 is a key mediator downstream of EZH2, and that disruption of an EZH2-miR-361-Twist regulatory axis may contribute to EC.

### GSK343 treatment mimics EZH2 knockdown effects on miR-361 and Twist expression

Because multiple epigenetic repression mechanisms, including EZH2-mediated histone methylation, DNA methylation, and histone deacetylase (HDAC)-induced hypoacetylation, are linked to gene silencing, we speculated that combinatorial use of epigenetic drugs targeting distinct epigenetic machinery might achieve a synergistic effect on activation of the silenced genes. The HDAC inhibitor, suberoylanilide hydroxamic acid (SAHA), stimulates EC cell migration [21]. We confirmed this effect in Ishikawa cells (Supplementary Figure 9A) and found that SAHA treatment promoted SPAC-1-L cell invasion (Supplementary Figure 9B). Previous reports showed that treatment with the DNA methylation inhibitor, 5-aza-2′-deoxycytidine (5-AZA), restored expression of miR-34b, a tumor suppressor that inhibits EC cell growth and invasion [8]. We observed a dose-dependent increase in miR-34b expression in SPAC-1-L and HOUA-I cells treated with 5-AZA (Supplementary Figure 10). 5-AZA treatment also induced miR-361 expression (Supplementary Figure 10), indicating that miR-361 silencing in EC cells is mediated, at least in part, by DNA methylation.

Using a computational bioinformatics analysis, we screened CpG islands upstream of *miR-361*, but detected no CpG-enriched region (data not shown), indicating that miR-361 induction by 5-AZA might result from epigenetic activation of an upstream miR-361 regulator. We then investigated the antineoplastic effects of GSK343 (a specific inhibitor of EZH2 methyltransferase activity) [22] combined with 5-AZA.

**Figure 4:**
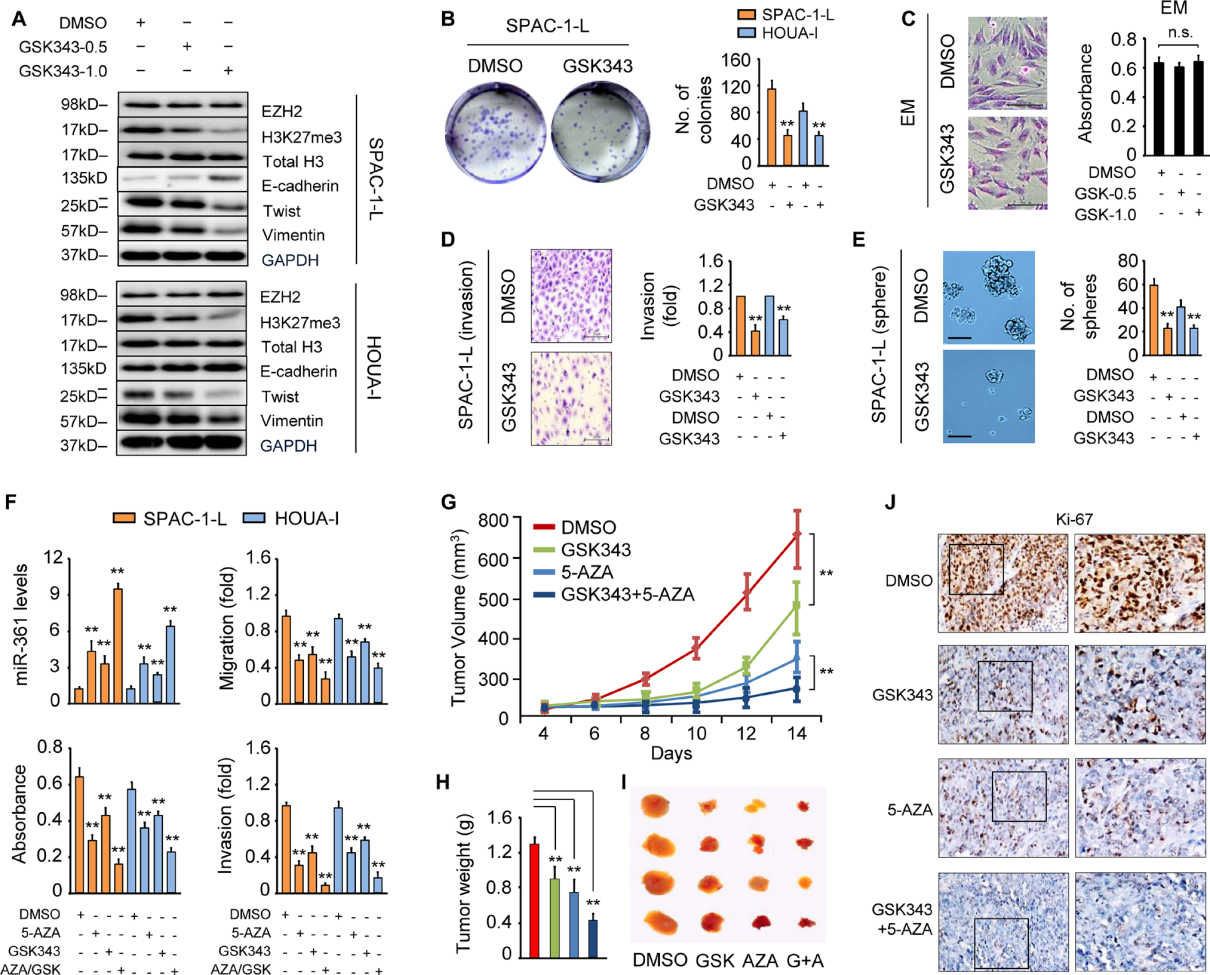
GSK343 mimics the effects of EZH2 knockdown on miR-361 and Twist expression (**A**) Western blot analysis in SPAC-1-L (upper) and HOUA-I (lower) cells treated with or without GSK343 (0.5 or 1 μM, 72 h). (**B**) Clone formation assays with SPAC-1-L and HOUA-I cells treated with GSK343 (1 μM, 14 d) exhibit reduced colony formation. (**C**) Images of immortalized endometrial epithelial cell EM (scale bar: 100 μm) stained with Giemsa after 72 h of exposure to DMSO or GSK343 (1 μM, 72 h) (left). Viability of EM cells treated with GSK343 (0.5 or 1 μM) was determined by CCK-8 assay (right). n.s., not significant. (**D**) Relative invasion of SPAC-1-L and HOUA-I cells treated with or without GSK343 (1 μM, 72 h). Data are presented as fold change over DMSO-treated cells. (**E**) GSK343 treatment (1 μM) decreases sphere formation by SPAC-1-L and HOUA-I cells. Representative images of spheres (scale bar: 100 μm) taken at 14 d. (**F**) miR-361 expression, proliferation, migration and invasion in SPAC-1-L and HOUA-I cells treated with 5-AZA (10 μM) and/or GSK343 (1 μM) for 72 h. Measurement of Ishikawa-derived xenograft tumors after treatment with GSK343 and 5-AZA alone or in combination. Tumor volume (**G**) and tumor weight (**H**) are shown (*n* = 4/group). (**I**) Representative images of tumor samples for each treatment group endpoint. (**J**) Immunohistochemistry for Ki-67 expression in tumors from mice treated as indicated. ^*^P < 0.01.

Western blot analysis demonstrated that GSK343 treatment dose-dependently reduced levels of H3K27Me3, the enzymatic product of EZH2 methyltransferase, without affecting total H3. GSK343 had no effect on EZH2 expression in SPAC-1-L and HOUA-I cells (Figure 4A). GSK343 treatment also reduced EC cell proliferation as measured by clone formation assay (Figure 4B), and impaired invasion and sphere formation (Figure 4D–4E). However, it did not affect cellular morphology or viability in normal endometrial epithelial EM cells (Figure 4C).

We then surveyed miR-361 and EMT-related marker expression. Similar to EZH2 knockdown, GSK343 treatment enhanced miR-361 and E-cadherin levels, and decreased the expression of Snail, N-cadherin, Twist and Vimentin (Figure 4A, 4F and Supplementary Figure 8F). EZH2 enzymatic activity inhibition may provide an option for treatment of EC or other tumors. Finally, we analyzed whether the combination of GSK343 and 5-AZA would have synergistic antitumor activity. As single agents, GSK343 and 5-AZA suppress EC cell proliferation, migration and invasion, but their combination synergistically upregulated miR-361 and enhanced inhibition of cell growth, migration and invasion as compared to either agent alone (Figure 4F).

Based on our *in vitro* results, we evaluated the effects of GSK343 and 5-AZA alone and combined on EC growth *in vivo*. Tumors generated by inoculation of Ishikawa cells were treated with the vehicle, GSK343, 5-AZA, or GSK343 plus 5-AZA. As single agents, GSK343 and 5-AZA decreased tumor size and weight compared to vehicle alone, and when combined, increased tumor growth inhibition compared to either agent alone (Figure 4G–4I). Consistent with tumor growth inhibition, immunohistochemical analysis demonstrated that combined treatment suppressed the proliferation marker, Ki-67, as compared to either agent alone (Figure 4J). Together, these data suggest that GSK343 prevents EC growth *in vivo* and the combined use of GSK343 and 5-AZA shows potential as a novel anti-cancer therapy.

### Clinical relevance of the EZH2–miR-361-Twist axis in EC

To establish the clinical significance of altered miR-361 expression in EC progression, we examined the association between miR-361 expression and clinicopathologic variables. We divided 24 EC patients into two groups: those with lower than average miR-361 expression (*n* = 12) and those with higher than average miR-361 expression (*n* = 12). Reduced miR-361 was associated with poorly differentiated histology (Supplementary Table 3), indicating that miR-361 repression is important for EC growth and/or progression.

**Figure 5:**
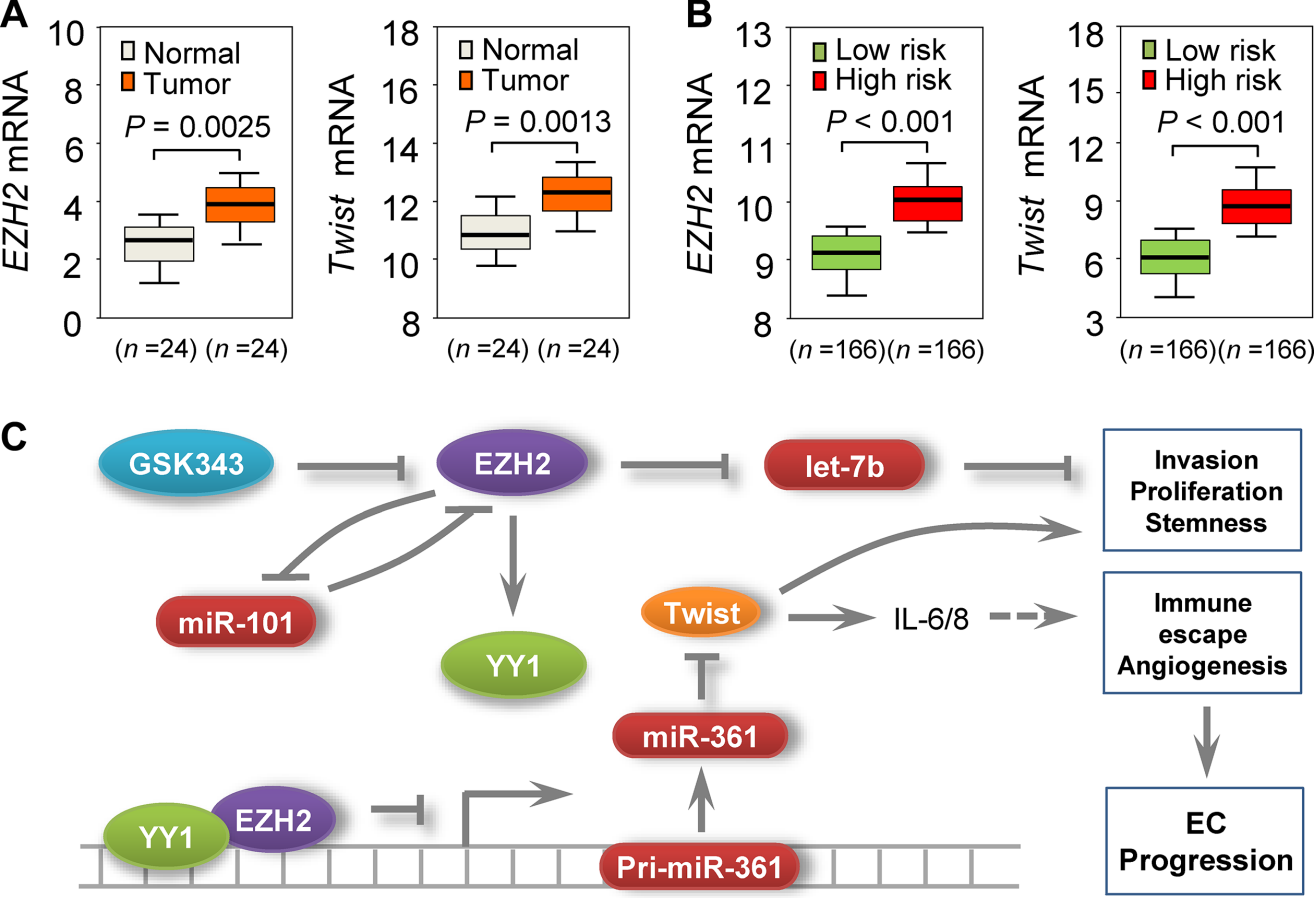
Clinical relevance of the EZH2–miR-361-Twist axis in EC (**A**) qPCR analysis of *EZH2* or *Twist* in 24 EC and adjacent normal samples. (**B**) TCGA database-extracted data showing higher *EZH2* and *Twist* expression in EC patients with high risk of poor survival. (**C**) Proposed mechanism involving an EZH2-miR-361-Twist axis to regulate EC progression. miR-101 downregulation leads to overexpression of its negative feedback regulator, EZH2, which acts as a co-suppressor of YY1 to epigenetically suppress miR-361, upregulating Twist (a direct target of miR-361). Twist upregulation promotes EC cell invasion, proliferation, and cancer stemness, and is associated with increased IL-6/8 expression. EZH2 also silences let-7b expression and contributes to EC development. EZH2-specific inhibitor GSK343 mimics the effects of EZH2 knockdown on miR-361 and Twist expression.

We then assessed *EZH2* and *Twist* expression in EC and adjacent normal tissues using qRT-PCR. *EZH2* and *Twist* levels were increased in EC samples (Figure 5A), while miR-361 was downregulated (Figure 1C). Subsequent meta-analysis using TCGA database showed that increased expression of *EZH2* or *Twist* was associated with poorer prognosis in EC patients (Figure 5B), suggesting an inverse correlation between EZH2/Twist and miR-361 expression in EC. Our *in vivo* results corroborated our *in vitro* data and support the notion that epigenetic silencing of miR-361 by EZH2 upregulates Twist expression in EC.

## DISCUSSION

Dissecting the molecular pathways that drive EC invasion and metastasis is crucial to the development of novel anti-tumor therapies and for improving patient survival. Here, we analyze EZH2-mediated transcriptional repression in EC cells and describe previously unrecognized functional interactions between EZH2, miR-361, and Twist. Our results suggest that this signaling pathway plays an important role in EC progression. We demonstrated that miR-361 is a tumor suppressor in EC, and EZH2 binds directly to the miR-361 promoter to suppress its transcription through a YY1-dependent manner. This upregulates Twist, a direct target of miR-361 in EC cells (Figure 5C).

In agreement with previous studies showing that numerous miRNAs are silenced by EZH2 in human cancer cells [7, 16], in EC cells, EZH2 mediated suppression of let-7b and miR-361, both of which inhibit EC cell proliferation, invasion and self-renewal. These effects were partially mediated by restoration of the epithelial phenotype and inhibition of PI3K/AKT signaling. Our clinical data combined with TCGA dataset analysis also associated reduced let-7b and miR-361 levels with worse patient survival rates, implicating these miRNAs as potential tumor suppressors across diverse cancer types. miR-101 repression upregulates expression of its target, EZH2 [6], and EZH2 inhibition in EC cells induces miR-101 expression (Figure 3C), suggesting a miR-101/EZH2 double-negative feedback loop.

Our results suggest that reactivating tumor suppressor miRNAs by targeting EZH2 may be a promising approach for EC treatment. However, epigenetic changes, such as DNA methylation, are also implicated in EC development [1]. miR-34b silencing in EC cells through DNA methylation can be recovered via 5-AZA treatment [8], although 5-AZA cannot reactivate all genes silenced by methylation, possibly due to retention of the repressive histone marker, H3K27me3 [23]. These data highlight the need to target multiple epigenetic abnormalities through the combined use of an EZH2 inhibitor and 5-AZA. We found that EZH2 inhibition by GSK343 effectively restored miR-361 expression and phenocopied the effects of EZH2 knockdown *in vitro*. GSK343 plus 5-AZA synergistically activated miR-361 and attenuated tumor cell metastatic potential. Takeshima, *et al*. [24] also found that GSK126 plus 5-AZA produced additive antitumor effects. Thus, patients with EZH2-driven EC by may benefit from the combined use of GSK343 (or other specific EZH2 inhibitors) and 5-AZA.

Novel therapies that target the tumor microenvironment rather than tumor cells themselves are of increasing interest. Twist promotes EMT and simultaneously regulates various genes involved in angiogenesis, inflammation, and the anti-tumor immune response [11–13]. To our knowledge, this is the first report linking the EMT process triggered by EZH2-miR-361-Twist signaling to a gene cluster that contains various cytokines (IL-6/8 and IFN-α/γ) and an angiogenic factor (VEGFA). Our data are consistent with previous findings that, in cancer cells, EZH2 mediates genes implicated in immunity, inflammation, and angiogenesis [25, 26]. Together, our results identify a pathway, EZH2-miR-361-Twist, that positive regulates tumor malignancy by promoting cancer cell proliferation, invasion, and self-renewal, and further support EZH2 as a promising therapeutic anti-EC target.

## MATERIALS AND METHODS

### Cell culture, reagents and transient transfection

The human serous EC cell line, SPAC-1-L, was obtained from Dr. Fumihiko Suzuki (Tohoku University, Sendai, Japan) and maintained in RPMI-1640 medium (Sigma-Aldrich, St. Louis, MO) supplemented with 10% fetal bovine serum (FBS). The EC cell lines, Ishikawa (JCRB Cell Bank, Osaka, Japan) and HOUA-I (RIKEN cell bank, Tsukuba, Japan), were grown in DMEM/F12 (Sigma-Aldrich, St. Louis, MO, USA) supplemented with 10% FBS. The immortalized human endometrial epithelial cell line, EM, was obtained from Professor Satoru Kyo (Shimane University, Japan) and cultured in DMEM/F12 supplemented with 15% FBS. Cells were seeded and incubated for 1 d, then treated with GSK343 (0.5 or 1 μM, ApexBio Technology, TX, USA; A3449) and/or 5-AZA (10 μM, Sigma-Aldrich, MO, USA; A3656) for 3 or 14 d as indicated. Media was changed daily or added every 3 d (sphere formation assay). Transient transfection of miRNA mimic, antisense miRNA inhibitor, and siRNA (Ambion, Austin, TX), or plasmids including pCMV6-AC, pCMV6-TWIST (321467; OriGene, Rockville, MD), pCMV/hygro and pCMV/hygro-EZH2 (11337; Sino Biological, Beijing, China), was accomplished using Lipofectamine 2000 (Invitrogen, Carlsbad, CA), according to the manufacturer's instructions.

### Quantitative real time RT-PCR analysis

Total RNA was extracted using TRIzol reagent (Invitrogen, Carlsbad, CA), and was reverse-transcribed into cDNA using the PrimeScript RT reagent kit (TaKaRa, Japan) according to the manufacturer's instructions. miRNA and mRNA qRT-PCR was performed using NCode miRNA qRT-PCR analysis (Invitrogen, Carlsbad, CA) and Takara SYBR Premix Ex Taq II (Takara, Japan), respectively. Forward primers for miRNA detection were exact sequences of mature miRNAs. GAPDH was used for normalization. Primers used for mRNA expression were obtained from the PrimerBank database (http://pga.mgh.harvard.edu/primerbank/).

### Cell viability, proliferation, and colony formation assays

Cells (5×10^3^) were plated in 96-well plates for 24 h and then treated with DMSO or varying doses of paclitaxel (Cell Signaling Technology, Beverly, MA). After 24 h, cell viability was determined using the Cell Counting Kit-8 (Dojindo, Kumamoto, Japan). Absorbance was determined at 450 nm using a microplate reader, and percent absorbance was calculated against DMSO-treated cells. Cells were transfected or treated as indicated for 72 h, and cell proliferation was assessed using the Cell Counting Kit-8. In the colony formation assay, approximately 500 cells were added to each well of a 6-well culture plate. Each experiment was performed in triplicate. After 14 d of culture at 37°C, cells were fixed using 10% formalin and then stained using 10% Giemsa. Colonies containing ≥50 cells were counted under a microscope.

### Cell migration, invasion and wound healing assays

Cell invasion and migration were monitored and analyzed as described previously [27, 28]. 5 × 10^4^ cells resuspended in serum-free medium were added to the upper inserts. In the lower chamber, 750 μl medium supplemented with 10% FBS served as a chemoattractant. After incubation for 24 h, cells adhering to the lower membrane surface were counted under a microscope. Migration assays were carried out in the same way as the invasion assay, except that the membrane was not coated with matrigel, and the incubation time was 12 h. For the wound-healing assay, cells were seeded in a 6-well plate. At confluence, wounds were carefully made using a 200-μl pipette tip, and cells were incubated with fresh medium containing Mitomycin C (5 μg ml^-1^) for 12 h. Distance migrated was quantified using pictures taken at 0 and 12 h.

### Western blotting

Whole-cell or nuclear protein extracts were prepared using the M-Per Mammalian Protein Extraction Reagent (Pierce Biotechnology, Woburn, MA) or the Nuclear Extraction Kit (Chemicon International, Temecula, CA) according to the manufacturer's instructions. Total protein (30 μg) and nuclear protein (10 μg) were loaded onto 10–20% SDS-PAGE gels, electrophoresed, and then transferred to nitrocellulose membranes. Antigen-antibody complexes were detected using the enhanced chemiluminescence blotting analysis system (Amersham Pharmacia Biotech, Buckinghamshire, UK). The following antibodies were used: EZH2 (Cell Signaling; 5246), H3K27me3 (Cell Signaling; 9733), total histone 3 (Cell Signaling; 9715), E-cadherin (GenScript; A01589), Vimentin (GenScript; A01189), Twist (Abcam; ab50887), p-AKT (Santa Cruz; sc-293125), AKT (Santa Cruz; sc-1618), GAPDH (Santa Cruz; sc-47724), YY1 (Santa Cruz; sc-7341) and lamin B1 (Santa Cruz; sc-20682). GAPDH (whole cell lysate) and lamin B (nuclear protein) were applied as loading controls. Primary antibodies were used at a dilution of 1:1000.

### Sphere formation assay

Cells (1000 ml^-1^) were cultured in serum-free medium supplemented with N2 plus media supplement (Invitrogen, CA), epidermal growth factor (20 ng ml^-1^, Invitrogen, CA), basic fibroblast growth factor (20 ng ml^-1^, Invitrogen, CA), and heparin (4 mg ml^-1^, Sigma-Aldrich, UK) for 14 d. Spheres > 50 μm were counted.

### Microarray experiments

RNA was purified from SPAC-1-L cells transfected with miR-101 or control mimic. The miRNA expression profile was assessed using a Superprint G3 Human GE 8 × 60k Microarray (Agilent Technologies) as previously described [6]. *P* < 0.05 represented differentially expressed miRNAs.

### Luciferase activity assay

*Twist* 3′-UTR luciferase vectors were obtained from OriGene (sc209156). To construct pri-miR-361 promoter luciferase reporter vectors, the promoter region encompassing YY1 binding sites (2, 5 or 8) was amplified from human genomic DNA and cloned into pGL3 vector *MluI*/*BglII* sites (Promega). A quick-change site-directed mutagenesis kit (Stratagene, CA) was used to mutate the miR-361 binding site within the *Twist* 3′-UTR or YY1 binding sites on the miR-361 promoter. Luciferase activity was measured 24 h after transfection using the dual-luciferase reporter assay system (Promega, WI). The Renilla luciferase reporter plasmid, pRL-CMV, was used to normalize transfection efficiency, and firefly luciferase activity was normalized to Renilla luciferase activity. Primers are provided in Supplementary Table 2.

### ChIP-qPCR assays

Chromatin from EC cells was immunoprecipitated with antibodies against EZH2 (Cell Signaling), H3K27me3 (Cell Signaling), YY1 (Santa Cruz), or IgG (Santa Cruz) as a control, using the Pierce Agarose ChIP kit (Pierce; Thermo Scientific, Rockford, IL, USA) as previously described [3]. Immunoprecipitated DNA was quantified using Takara SYBR Premix Ex Taq II (Takara, Japan). Results were expressed as fold enrichment over IgG control, and were further normalized to the *GAPDH* promoter (negative control). The human let-7a [17] promoter was used as a positive control for YY1 and EZH2 binding. Primers used for the ChIP assay are provided in Supplementary Table 2.

### Xenograft experiments and immunohistochemistry analysis

All experiments involving mice were performed in accordance with the guidelines of the animal care and use committee of the Cancer Center, Sun Yat-Sen University. Ishikawa cells (1×10^6^) were suspended in phosphate-buffered saline (PBS; 100 μl) and then injected subcutaneously into five-week-old female nude mice (BALB/c). When tumors became palpable, (i.e., about approximately 70–80 mm^3^), mice were intraperitoneally injected with control vehicle (DMSO), 5-AZA (0.2 mg kg^-1^) and/or GSK343 (15 mg kg^-1^) three times weekly for 2 weeks, after which mice were sacrificed. Tumor length and width were measured using calipers, and tumor volume was calculated using the formula: tumor volume = length × width^2^ × 0.5. In a parallel experiment, portions of excised tumors embedded in paraffin were subjected to immunohistochemical analysis for Ki-67 (Abcam; ab15580) expression as previously described [6].

### Paired tumor and non-tumor tissues

Twenty-four pairs of EC and adjacent non-tumor endometrial tissues were used in this study following review and approval by the Cancer Center, Sun Yat-Sen University. Informed consent was obtained from all patients before operation. Clinical and pathological data are described in Supplementary Table 3. Samples were immediately snap-frozen at -80°C, and total RNA was isolated using TRIzol reagent.

### Statistical analysis

All experiments were performed in triplicate. Results are expressed as means ± SEMs, and 2-tailed Student's *t*-tests were used for statistical analysis. Fisher exact tests were used to compare categorical data. *P* < 0.05 represented statistical significance.

## SUPPLEMENTARY MATERIALS FIGURES AND TABLES



## References

[1] Dong P, Kaneuchi M, Konno Y, Watari H, Sudo S, Sakuragi N (2013). Emerging therapeutic biomarkers in endometrial cancer. Biomed Res Int.

[2] Dong P, Kaneuchi M, Watari H, Sudo S, Sakuragi N (2014). MicroRNA-106b modulates epithelial–mesenchymal transition by targeting TWIST1 in invasive endometrial cancer cell lines. Mol Carcinog.

[3] Dong P, Karaayvaz M, Jia N, Kaneuchi M, Hamada J, Watari H, Sudo S, Ju J, Sakuragi N (2013). Mutant p53 gain-of-function induces epithelial-mesenchymal transition through modulation of the miR-130b-ZEB1 axis. Oncogene.

[4] Dong P, Kaneuchi M, Watari H, Hamada J, Sudo S, Ju J, Sakuragi N (2011). MicroRNA-194 inhibits epithelial to mesenchymal transition of endometrial cancer cells by targeting oncogene BMI-1. Mol Cancer.

[5] Yamaguchi H, Hung MC (2014). Regulation and Role of EZH2 in Cancer. Cancer Res Treat.

[6] Konno Y, Dong P, Xiong Y, Suzuki F, Lu J, Cai M, Watari H, Mitamura T, Hosaka M, Hanley SJ, Kudo M, Sakuragi N (2014). MicroRNA-101 targets EZH2, MCL-1 and FOS to suppress proliferation, invasion and stem cell-like phenotype of aggressive endometrial cancer cells. Oncotarget.

[7] Cao Q, Mani RS, Ateeq B, Dhanasekaran SM, Asangani IA, Prensner JR, Kim JH, Brenner JC, Jing X, Cao X, Wang R, Li Y, Dahiya A (2011). Coordinated regulation of polycomb group complexes through microRNAs in cancer. Cancer Cell.

[8] Hiroki E, Suzuki F, Akahira J, Nagase S, Ito K, Sugawara J, Miki Y, Suzuki T, Sasano H, Yaegashi N (2012). MicroRNA-34b functions as a potential tumor suppressor in endometrial serous adenocarcinoma. Int J Cancer.

[9] Kent OA, Mendell JT (2006). A small piece in the cancer puzzle: microRNAs as tumor suppressors and oncogenes. Oncogene.

[10] Kanitz A, Imig J, Dziunycz PJ, Primorac A, Galgano A, Hofbauer GF, Gerber AP, Detmar M (2012). The expression levels of microRNA-361-5p and its target VEGFA are inversely correlated in human cutaneous squamous cell carcinoma. PLoS One.

[11] Palena C, Hamilton DH, Fernando RI (2012). Influence of IL-8 on the epithelial–mesenchymal transition and the tumor microenvironment. Future Oncol.

[12] Mironchik Y, Winnard PT, Vesuna F, Kato Y, Wildes F, Pathak AP, Kominsky S, Artemov D, Bhujwalla Z, Van Diest P, Burger H, Glackin C, Raman V (2005). Twist overexpression induces in vivo angiogenesis and correlates with chromosomal instability in breast cancer. Cancer Res.

[13] Li S, Kendall SE, Raices R, Finlay J, Covarrubias M, Liu Z, Lowe G, Lin YH, Teh YH, Leigh V, Dhillon S, Flanagan S, Aboody KS (2012). TWIST1 associates with NF-κB subunit RELA via carboxyl-terminal WR domain to promote cell autonomous invasion through IL8 production. BMC Biol.

[14] Niesner U, Albrecht I, Janke M, Doebis C, Loddenkemper C, Lexberg MH, Eulenburg K, Kreher S, Koeck J, Baumgrass R, Bonhagen K, Kamradt T, Enghard P (2008). Autoregulation of Th1-mediated inflammation by twist1. J Exp Med.

[15] Lee Sylvia, Margolin Kim (2011). Cytokines in cancer immunotherapy. Cancers (Basel).

[16] Bao B, Ali S, Banerjee S, Wang Z, Logna F, Azmi AS, Kong D, Ahmad A, Li Y, Padhye S, Sarkar FH (2012). Curcumin analogue CDF inhibits pancreatic tumor growth by switching on suppressor microRNAs and attenuating EZH2 expression. Cancer Res.

[17] Chen Y, Jacamo R, Konopleva M, Garzon R, Croce C, Andreeff M (2013). CXCR4 downregulation of let-7a drives chemoresistance in acute myeloid leukemia. J Clin Invest.

[18] Wang H, Garzon R, Sun H, Ladner KJ, Singh R, Dahlman J, Cheng A, Hall BM, Qualman SJ, Chandler DS, Croce CM, Guttridge DC (2008). NF-kappaB-YY1-miR-29 regulatory circuitry in skeletal myogenesis and rhabdomyosarcoma. Cancer Cell.

[19] Zhang X, Zhao X, Fiskus W, Lin J, Lwin T, Rao R, Zhang Y, Chan JC, Fu K, Marquez VE, Chen-Kiang S, Moscinski LC, Seto E (2012). Coordinated silencing of MYC-mediated miR-29 by HDAC3 and EZH2 as a therapeutic target of histone modification in aggressive B-Cell lymphomas. Cancer Cell.

[20] Yang Y, Zhou L, Lu L, Wang L, Li X, Jiang P, Chan LK, Zhang T, Yu J, Kwong J, Cheung TH, Chung T, Mak K (2013). A novel miR-193a-5p-YY1-APC regulatory axis in human endometrioid endometrial adenocarcinoma. Oncogene.

[21] Uchida H, Maruyama T, Ono M, Ohta K, Kajitani T, Masuda H, Nagashima T, Arase T, Asada H, Yoshimura Y (2007). Histone deacetylase inhibitors stimulate cell migration in human endometrial adenocarcinoma cells through up-regulation of glycodelin. Endocrinology.

[22] Verma SK, Tian X, LaFrance LV, Duquenne C, Suarez DP, Newlander KA, Romeril SP, Burgess JL, Grant SW, Brackley JA, Graves AP, Scherzer DA, Shu A (2012). Identification of Potent, Selective, Cell-Active Inhibitors of the Histone Lysine Methyltransferase EZH2. ACS Med Chem Lett.

[23] McGarvey KM, Fahrner JA, Greene E, Martens J, Jenuwein T, Baylin SB (2006). Silenced tumor suppressor genes reactivated by DNA demethylation do not return to a fully euchromatic chromatin state. Cancer Res.

[24] Takeshima H, Wakabayashi M, Hattori N, Yamashita S, Ushijima T (2015). Identification of co-existence of DNA methylation and H3K27me3 specifically in cancer cells as a promising target for epigenetic therapy. Carcinogenesis.

[25] Sun F, Chan E, Wu Z, Yang X, Marquez VE, Yu Q (2009). Combinatorial pharmacological approaches target EZH2-mediated gene repression in breast cancer cells. Mol Cancer Ther.

[26] Crea F, Fornaro L, Bocci G, Sun L, Farrar WL, Falcone A, Danesi R (2012). EZH2 inhibition: targeting the crossroad of tumor invasion and angiogenesis. Cancer Metastasis Rev.

[27] Dong P, Xu Z, Jia N, Li D, Feng Y (2009). Elevated expression of p53 gain-of-function mutation R175H in endometrial cancer cells can increase the invasive phenotypes by activation of the EGFR/PI3K/AKT pathway. Mol Cancer.

[28] Dong PX, Jia N, Xu ZJ, Liu YT, Li DJ, Feng YJ (2008). Silencing of IQGAP1 by shRNA inhibits the invasion of ovarian carcinoma HO-8910PM cells in vitro. J Exp Clin Cancer Res.

