# Supplementary material for: EZH2 inhibition suppresses endometrial cancer progression via miR-361/Twist axis
Source: Oncotarget. 2017 Jan 10;8(8):13509–20. doi: 10.18632/oncotarget.14586 (PMC5355116; doi:10.18632/oncotarget.14586)
Supplement: Supplementary file 1 [file oncotarget-08-13509-s001.pdf]

EZH2 inhibition suppresses endometrial cancer progression via miR-361/Twist axis

Supplementary Materials

REFERENCES

1. Chen Y, Jacamo R, Konopleva M, Garzon R, Croce C, Andreeff M. CXCR4 downregulation of let-7a drives chemoresistance in acute myeloid leukemia. J Clin Invest. 2013; 123:2395–2407.

2. Yamagishi M, Nakano K, Miyake A, Yamochi T, Kagami Y, Tsutsumi A, Matsuda Y, Sato-Otsubo A, Muto S, Utsunomiya A, Yamaguchi K, Uchimaru K, Ogawa S, et al. Polycomb-mediated loss of miR-31 activates NIK-dependent NF-κB pathway in adult T cell leukemia and other cancers. Cancer Cell. 2012; 21:121–135.

Supplementary Table 1: Top 19 up-regulated and 15 down-regulated miRNAs by miR-101 mimic in human endometrial cancer SPAC-1-L cell, measured with microarray analysis

	Up-regulated miRNAs	
**No.**	**miRNA name**	**Fold change (miR-101/control mimic)**
1	hsa-miR-101	409.400
2	hsa-miR-371-5p	137.989
3	hsa-miR-429	129.773
4	hsa-miR-2276	123.795
5	hsa-miR-3935	120.434
6	hsa-miR-671-5p	118.236
7	hsa-miR-432	83.472
8	hsa-miR-424*	82.711
9	hsa-miR-622	79.592
10	hsa-miR-3654	64.650
11	hsa-miR-1972	59.724
12	hsa-miR-3610	58.056
13	hsa-miR-3156	56.923
14	hsa-miR-3125	56.555
15	hsa-miR-874	54.443
16	hsa-miR-296-5p	54.369
17	hsa-miR-665	36.375
18	hsa-miR-345	32.633
19	hsa-miR-1273e	30.746

	Down-regulated miRNAs	
**No.**	**miRNA name**	**Fold change (miR-101/control mimic)**
1	hsa-miR-212	0.008
2	hsa-miR-7	0.013
3	hsa-miR-424	0.013
4	hsa-let-7d	0.015
5	hsa-miR-31*	0.016
6	hsa-miR-455-3p	0.017
7	hsa-miR-96	0.017
8	hsa-miR-125b	0.018
9	hsa-miR-301a	0.018
10	hsa-miR-18a	0.019
11	hsa-miR-571	0.019
12	hsa-let-7g	0.327
13	hsa-miR-30b	0.329
14	hsa-miR-30c	0.400
15	hsa-miR-331-3p	0.413

Supplementary Table 2: Primers used for making Twist 3′-UTR-mutation luciferase constructs, pGL-3-miR-361 promoter luciferase reporter constructs and miR-361 promoter mutations (site 2, 5 and 8), or for ChIP-qPCR analysis

Twist 3′-UTR-mut F	CCTCACACCTCTGCATTCGACTAGAAGTCTGAACAGTTG	3′-UTR mutagenesis
Twist 3′-UTR-mut R	CAACTGTTCAGACTTCTAGTCGAATGCAGAGGTGTGAGG	3′-UTR mutagenesis
pGL-3-miR-361-2 F	CGCACGCGTCCCTCTGAAGTAGCGACCAG	Promoter luciferase reporter
pGL-3-miR-361-2 R	CCGAGATCTGCTGCAAAGCAACCTCTTTC	Promoter luciferase reporter
pGL-3-miR-361-M2 F	CTGGCTCCCGAAACCCGGGCTTTCTAGCCCACAG	mutagenesis
pGL-3-miR-361-M2 R	CTGTGGGCTAGAAAGCCCGGGTTTCGGGAGCCAG	mutagenesis
pGL-3-miR-361-5 F	CGCACGCGTCCACGTTTTCCTTGTCAG	Promoter luciferase reporter
pGL-3-miR-361-5 R	CCGAGATCTACCATAAAAACACATGTCCCAGT	Promoter luciferase reporter
pGL-3-miR-361-M5 F	ATTTGGGTTGATTCCCGCGCTTTGCTATTGTAAA	mutagenesis
pGL-3-miR-361-M5 R	TTTACAATAGCAAAGCGCGGGAATCAACCCAAAT	mutagenesis
pGL-3-miR-361-8 F	CGCACGCGTAAGCAGCTTGCTCAAAATTATA	Promoter luciferase reporter
pGL-3-miR-361-8 R	CGAGATCTTGTCCCATTTTGGGCATAGT	Promoter luciferase reporter
pGL-3-miR-361-M8 F	GAAAAGGAATCTCCCCGGGAATGAACTTATTCAG	mutagenesis
pGL-3-miR-361-M8 R	CTGAATAAGTTCATTCCCGGGGAGATTCCTTTTC	mutagenesis

miR-361-YY1-1 F	ATTCTGGGGCCTGGAGTATT	ChIP-qPCR
miR-361-YY1-1 R	CCCATGCAGAGTCCCTACTG	ChIP-qPCR
miR-361-YY1-2 F	GGATATGGGGAGCATGTTGT	ChIP-qPCR
miR-361-YY1-2 R	CAGGTCCTGTGGGCTAGAAA	ChIP-qPCR
miR-361-YY1-3 F	AAGAGGTTGCTTTGCAGCTC	ChIP-qPCR
miR-361-YY1-3 R	CTGGCCGTGTGGTAGAAAAG	ChIP-qPCR
miR-361-YY1-4 F	TGTCAGCATTTTGATCAGAGC	ChIP-qPCR
miR-361-YY1-4 R	CAGAAGAAGATAGTAAGATGAGGGAAA	ChIP-qPCR
miR-361-YY1-5 F	TTGATGAGCATTTGGGTTGA	ChIP-qPCR
miR-361-YY1-5 R	CATGTCCCAGTATGTTCACTGC	ChIP-qPCR
miR-361-YY1-6 F	TGGTAGAATGATTTGTATTCCTTTG	ChIP-qPCR
miR-361-YY1-6 R	CAGAACTACCATATGACCTAGCAA	ChIP-qPCR
miR-361-YY1-7 F	AGAGAAGGCCAAAAGTTACAA	ChIP-qPCR
miR-361-YY1-7 R	TCCCTCCATGTTGCTCCTAT	ChIP-qPCR
miR-361-YY1-8 F	CAACATGGAGGGAATGGAAA	ChIP-qPCR
miR-361-YY1-8 R	TGTCCCATTTTGGGCATAGT	ChIP-qPCR
miR-361-YY1-9 F	TGAACTTATTCAGTGATGACAGGA	ChIP-qPCR
miR-361-YY1-9 R	GGTTATGACAAAATCACAGTAGCA	ChIP-qPCR
miR-361-YY1-10 F	CATTGGTTTCCTCCCACAGT	ChIP-qPCR
miR-361-YY1-10 R	TCGAGCTTCACACACACACA	ChIP-qPCR
YY1-let-7a-3 pro F	GATGATGAAGTAGTTGGACCTT	ChIP-qPCR positive control (reference 1)
YY1-let-7a-3 pro R	CTGCCATTGAAATCTTGC	ChIP-qPCR positive control
GAPDH pro F	AACTTTCCCGCCTCTCAGC	ChIP-qPCR negative control (reference 2)
GAPDH pro R	CAGGAGGACTTTGGGAACGA	ChIP-qPCR negative control

Supplementary Table 3: The association between miR-361 expression and clinicopathologic factors of human endometrial cancers

Factors		miR-361 expression		*P*-value
		Low	High	
Grade	I-II	2	9	0.006
	III	10	3	
Tumor Diameter	≤ 3 cm	4	6	0.45
	> 3 cm	8	6	
Myometrial invasion	≤ 50%	7	10	0.22
	> 50%	5	2	
FIGO Stage	Ia	7	8	0.62
	Ib	2	3	
	II-III	3	1	
Lymph node metastasis	(−)	10	12	0.48
	(+)	2	0	
Ovarian metastasis	(−)	12	10	0.24
	(+)	0	2	

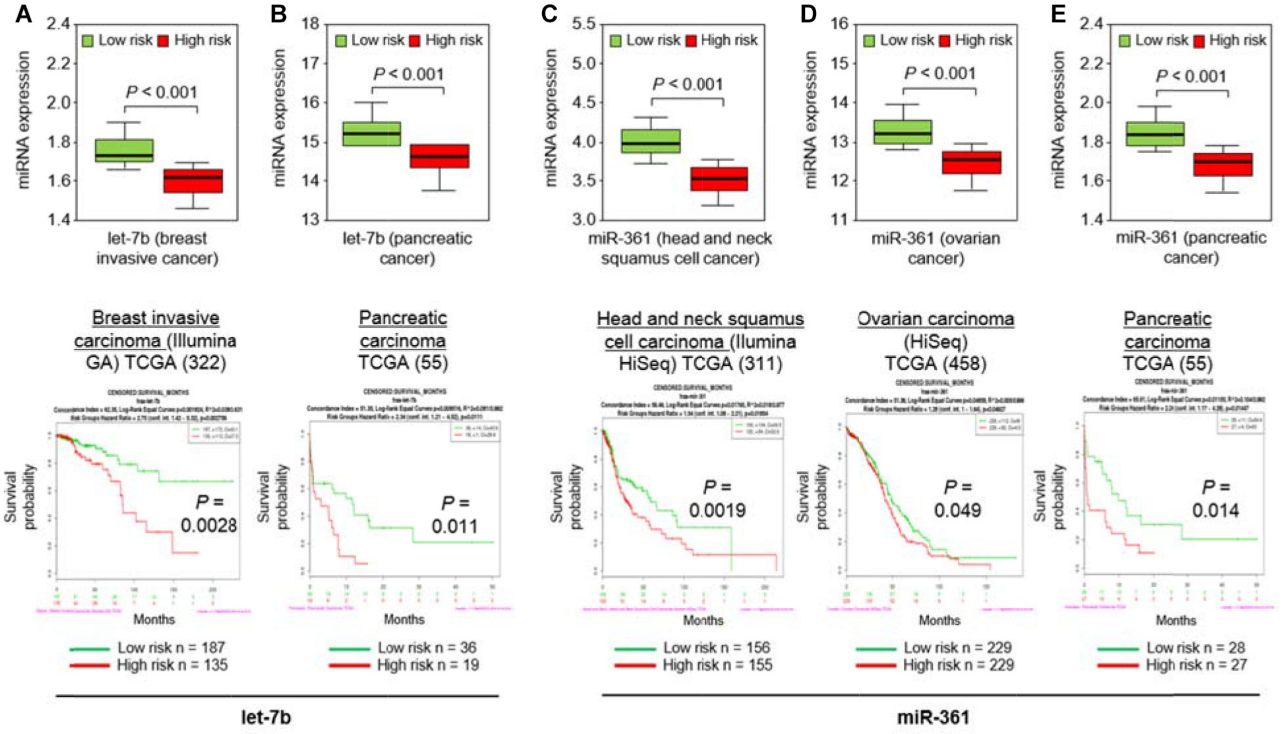

Supplementary Figure 1: Decreased let-7b and miR-361 levels are correlated with poor prognosis in several human tumors. Upper panel: The expression data of let-7b (**A** and **B**) or miR-361 (**C–E**) was extracted from the TCGA datasets comprising multiple cancer types and analyzed in SurvMicro database. Box plots demonstrating significantly lower levels of let-7b (a and b) or miR-361 (C–E) in high-risk patients. Lower panel: Kaplan-Meier plots of patients in multiple TCGA tumor datasets, which compare the overall survival between tumors with high-risk (red) or low-risk (green) of poor survival according to let-7b or miR-361-based prognostic scores were created using the SurvMicro database.

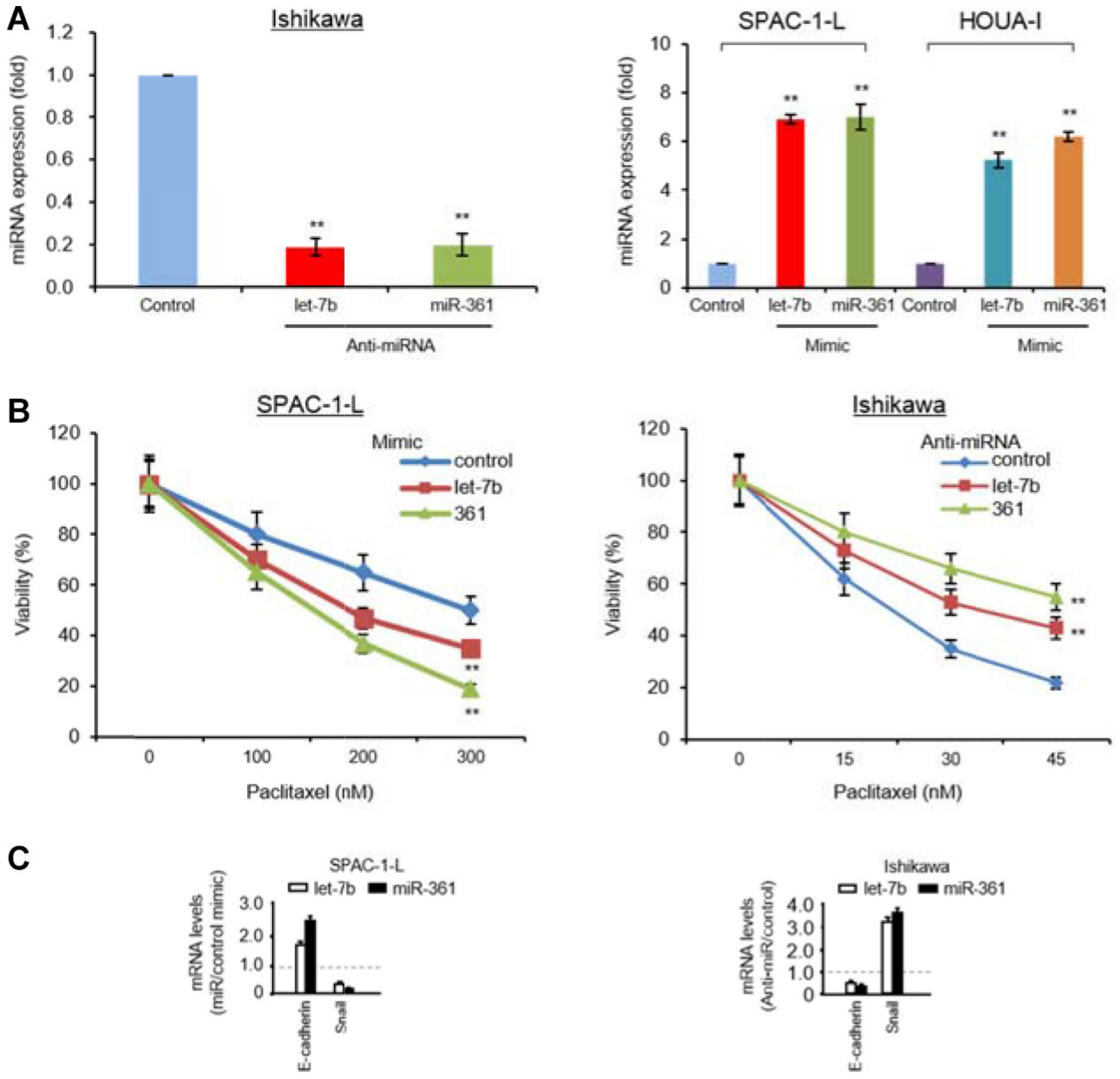

Supplementary Figure 2: Overexpression of miR-361 sensitizes EC cells to paclitaxel treatment. (**A**) qRT-PCR analysis of let-7b and miR-361 in Ishikawa cells transfected with anti-let-7b, anti-miR-361 or miRNA inhibitor negative control (left). let-7b and miR-361 levels were measured by qRT-PCRs in SPAC-1-L or HOUA-I cells after overexpression of let-7b or miR-361, respectively (right). (**B**) SPAC-1-L (left) and Ishikawa cells (right) were transfected with let-7b mimic, miR-361 mimic, anti-let-7b inhibitor, anti-miR-361 inhibitor or corresponding negative controls, followed by treatment with DMSO or with Paclitaxel for 24 hours. Cell viability was measured using cell counting kit-8 assay. The values were expressed as the percentage of viable cells, with the viability of DMSO-treated cells set at 100%. (**C**) qRT-PCR analysis of E-cadherin and Snail expression in SPAC-1-L cells transfected with let-7b mimic, miR-361 mimic or miRNA mimic negative control (left), and in Ishikawa cells transfected with anti-let-7b, anti-miR-361 or miRNA inhibitor negative control (right). $^{**}P < 0.01$.

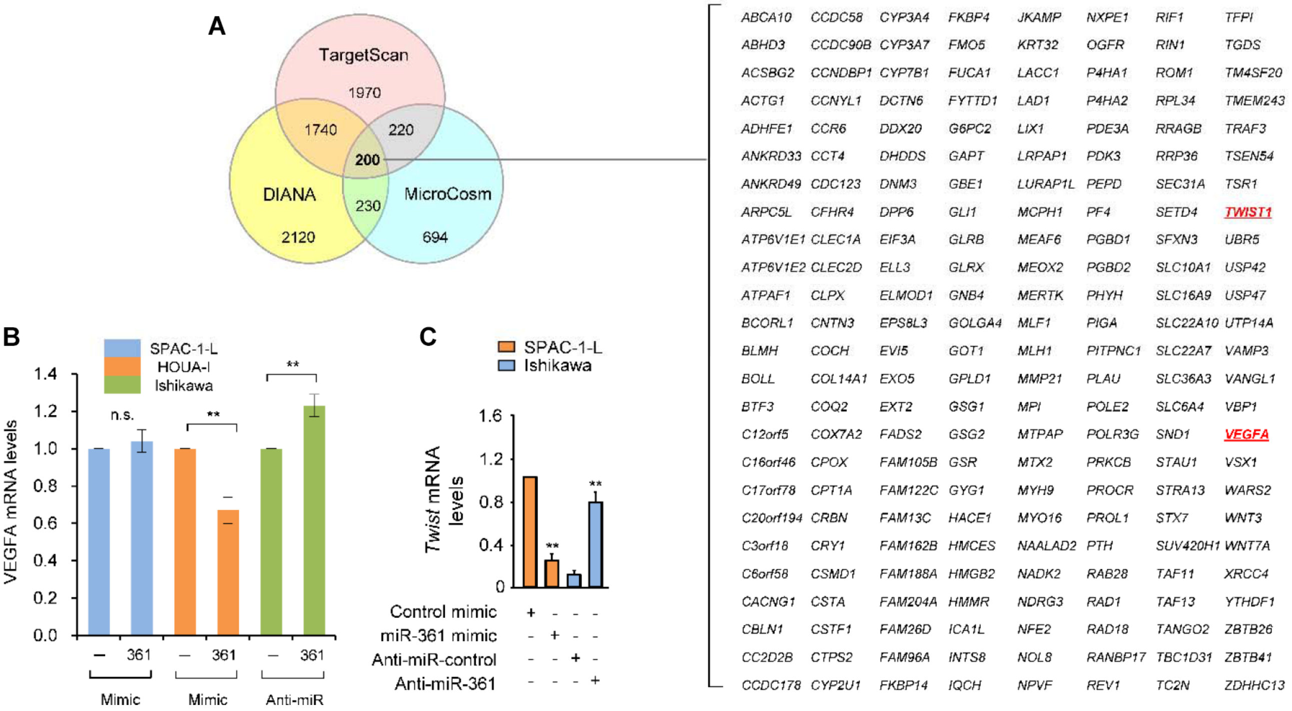

Supplementary Figure 3: Identification of genes that are candidate targets of miR-361 in EC cells. (**A**) Prediction algorithms (TargetScan, DIANA and MicroCosm) suggest that Twist1 and VEGFA, together with other 188 genes, might be regulated by miR-361 by direct targeting. (**B**) qRT-PCR analysis of VEGFA mRNA expression in three endometrial cancer cell lines transfected with miR-361 mimic, anti-miR-361 inhibitor or their negative controls as indicated. (**C**) Relative Twist mRNA expression in SPAC-1-L after overexpression of miR-361, or in Ishikawa cells upon knockdown of miR-361, using qRT-PCRs. n.s., not significant. $^{**}P < 0.01$.

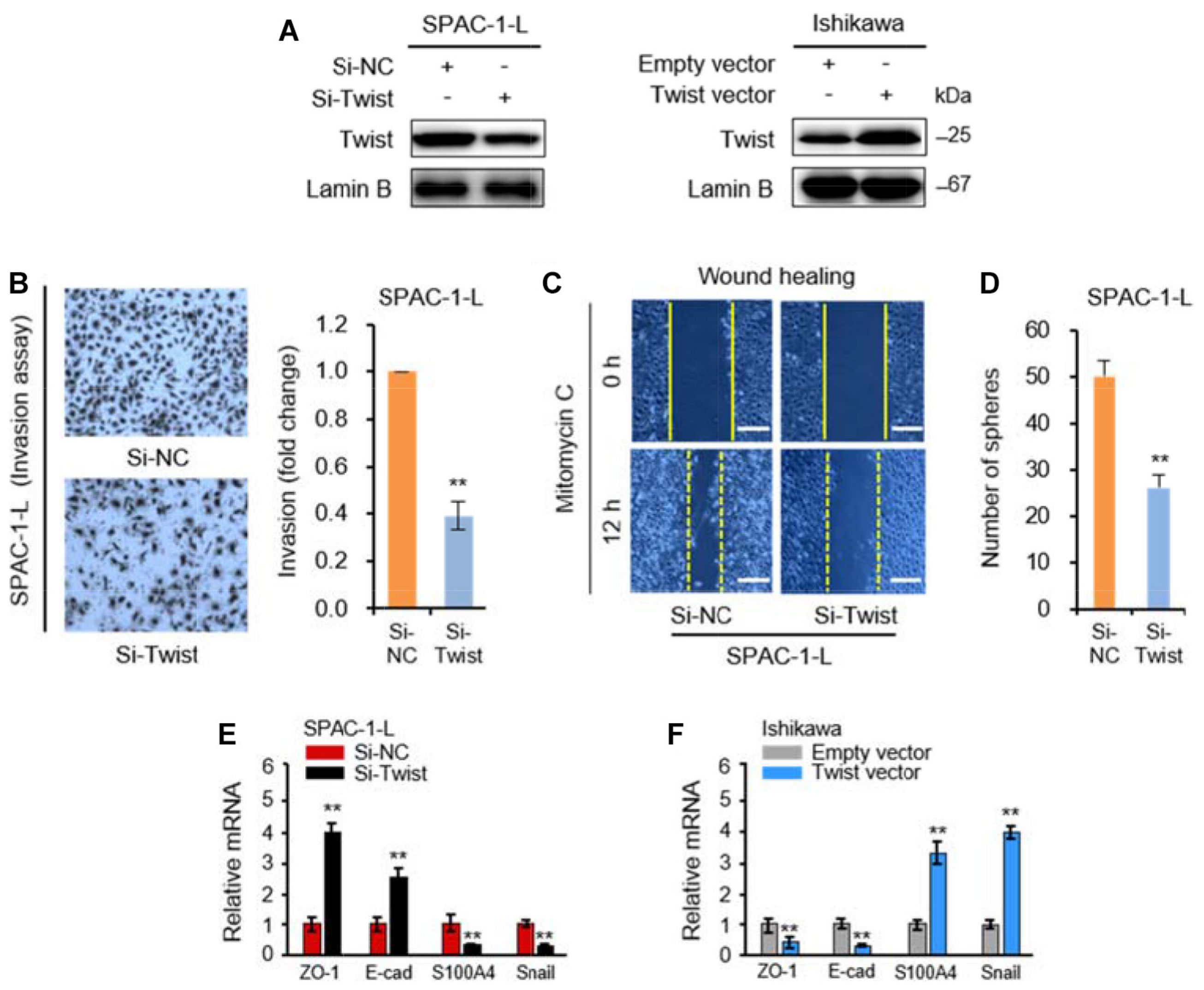

Supplementary Figure 4: Impact of Twist1 expression on EC cell invasion, migration and sphere formation. (**A**) Knockdown of Twist in SPAC-1-L cells (upper), or overexpression of Twist in Ishikawa (lower) cells and Twist protein expression was assessed using western blot analysis. (**B**) Cell invasion assay in SPAC-1-L cells treated with either Twist1 siRNA (Si-Twist) or negative control siRNA (Si-NC). Data are presented as fold change over Si-NC cells. (**C**) The scratch wound healing assay in SPAC-1-L cells transfected with either Si-Twist or Si-NC in the presence of Mitomycin C. Solid lines: the initial wound boundaries; dashed lines: the boundaries of migrated cells. (**D**) Sphere formation assay after transfection of SPAC-1-L cells with Si-NC or Si-Twist. SPAC-1-L (**E**) and Ishikawa (**F**) cells were transfected as indicated, and the mRNA levels of *ZO-1*, *E-cadherin*, *S100A4* and *Snail* in these cells were measured using qRT-PCRs. $^{**}P < 0.01$.

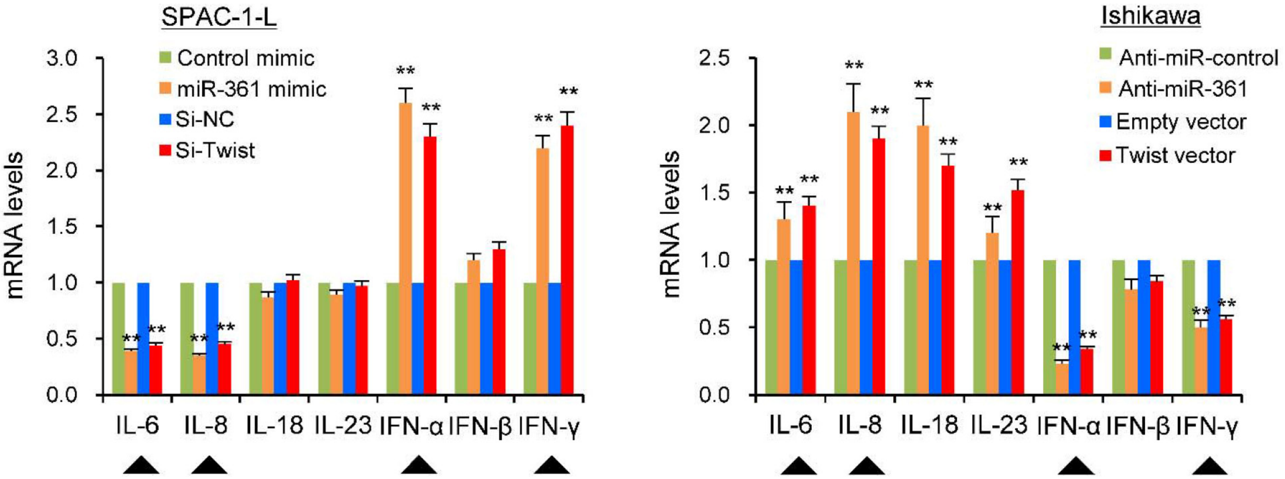

Supplementary Figure 5: Identification of Twist-regulated inflammatory genes in EC cells. SPAC-1-L (left) and Ishikawa (right) cells were transfected as indicated, and IL-6/8/18/23, IFN-α/β/γ mRNA levels in these cells were measured using qRT-PCR. Arrow heads mark those genes that are consistently regulated by miR-361 or Twist in both cell lines. $**P < 0.01$.

A

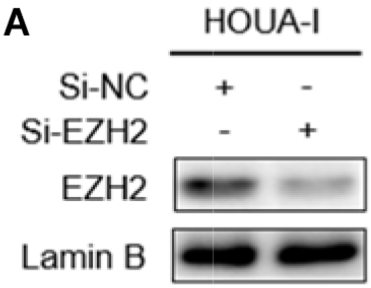

B

Transcription factors	No. of predicted binding site(s)
YY1	10
AP1	1
NF-Y	1
OCT1	2
HNF-4	2
USF-1	1
BRN2	1
v-Myb	1
HNF-3beta	1

Supplementary Figure 6: (**A**) Knockdown of EZH2 protein expression in HOUA-I cells, as assessed using western blot analysis. (**B**) TRANSFAC analysis identified numerous sites for various DNA-binding transcription factors upstream of pre-miR-361.

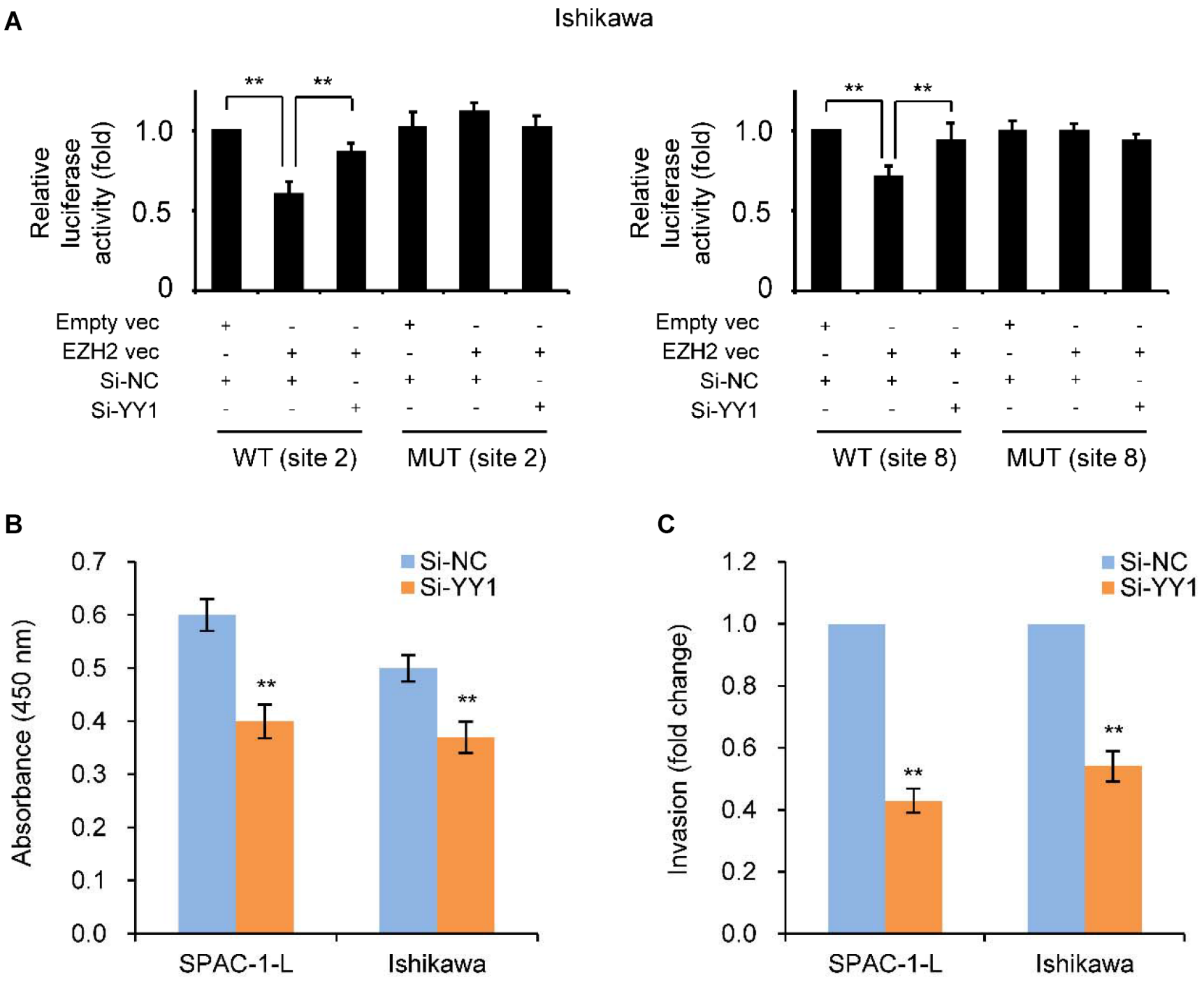

Supplementary Figure 7: Knockdown of YY1 abolished the miR-361 transcriptional repression induced by EZH2 and decreases cell proliferation and invasion of EC cells. Knockdown of YY1 abolished the miR-361 transcriptional repression (site 2 and 8) induced by EZH2 in Ishikawa cells (**A**), and decreases cell proliferation (**B**) and invasion (**C**) of SPAC-1-L and Ishikawa cells, as measured by reporter assays, cell counting kit-8 assay and invasion assay (data are presented as fold change over Si-NC cells), respectively. $^{**}P < 0.01$.

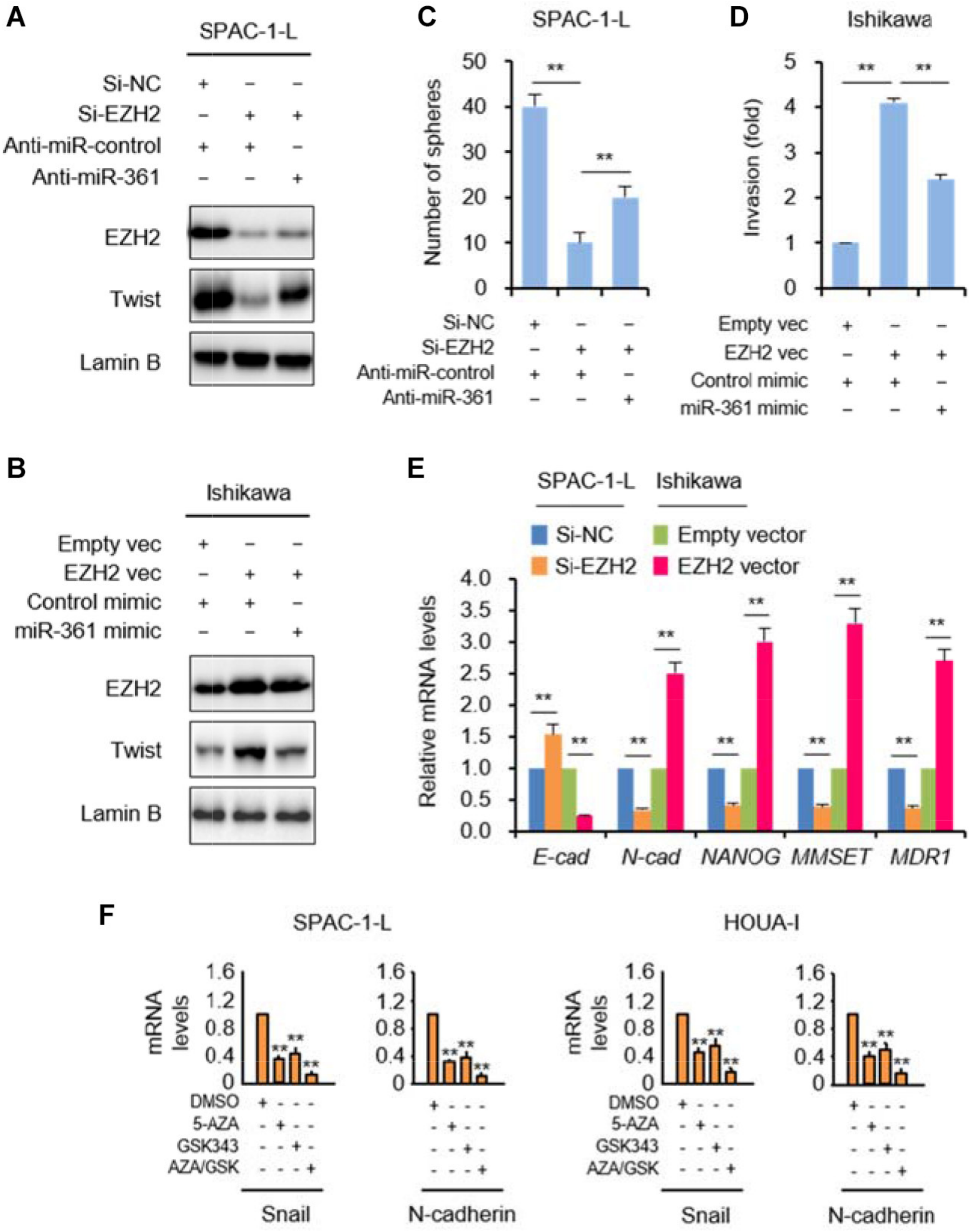

Supplementary Figure 8: EZH2-induced invasion and stemness requires miR-361 inhibition. EZH2, Twist, E-cadherin and Vimentin protein levels in SPAC-1-L cells transfected with Si-EZH2, together with or without anti-miR-361 inhibitor (**A**), or in Ishikawa cells transduced with EZH2 vector, together with or without miR-361 mimic (**B**). (**C**) Sphere formation assay of SPAC-1-L cells transfected with Si-EZH2, with or without anti-miR-361 inhibitor. (**D**) Invasion assay of Ishikawa cells transduced with EZH2 vector, with or without miR-361 mimic. (**E**) *E-cadherin*, *N-cadherin*, *NANOG*, *MMSET* and *MDR1* mRNA levels in SPAC-1-L cells transfected with Si-NC or Si-EZH2, or in Ishikawa cells transfected with empty vector or EZH2 vector. (**F**) Snail and N-cadherin mRNA levels in SPAC-1-L and HOUA-I cells treated with GSK343 and 5-AZA alone or in combination. $^{**}P < 0.01$.

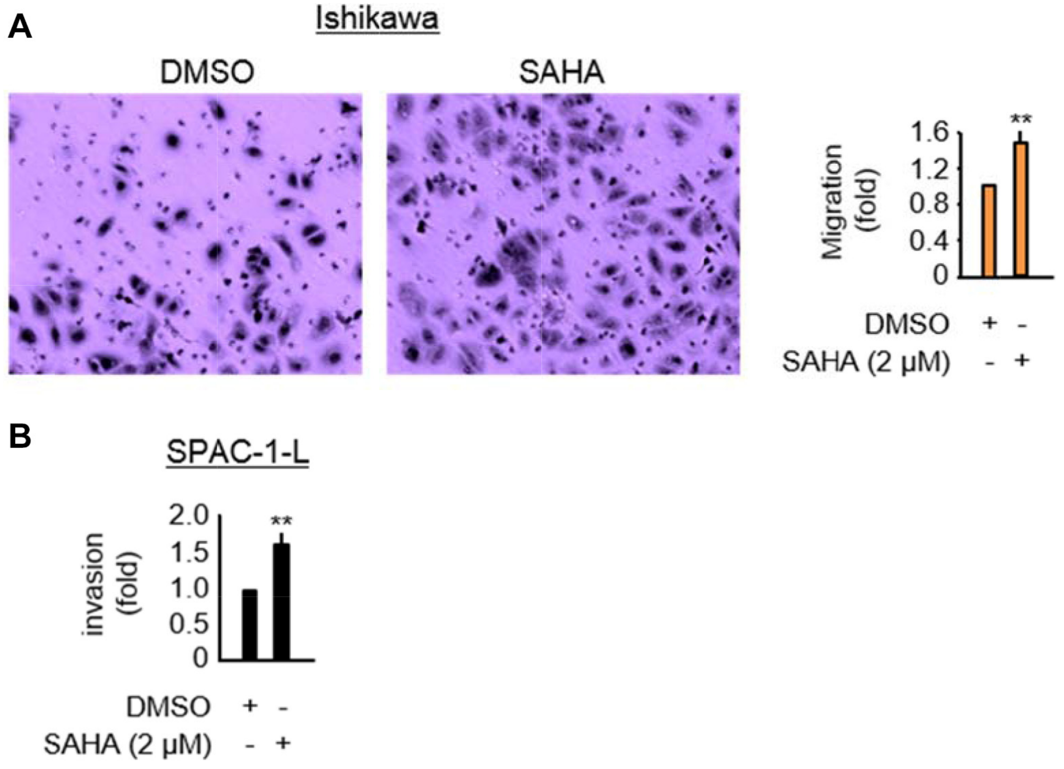

Supplementary Figure 9: The treatment with SAHA increased EC cell migration and invasion. (**A**) Transwell cell migration assay showing enhanced migration of Ishikawa cells treated with SAHA (2 µM) for 72 hours. (**B**) Cell invasion assays demonstrating that SAHA treatment (2 µM, 72 hours) promotes invasive potential of SPAC-1-L cells. Migration and invasion data are presented as fold change over DMSO-treated cells. $^{**}P < 0.01$.

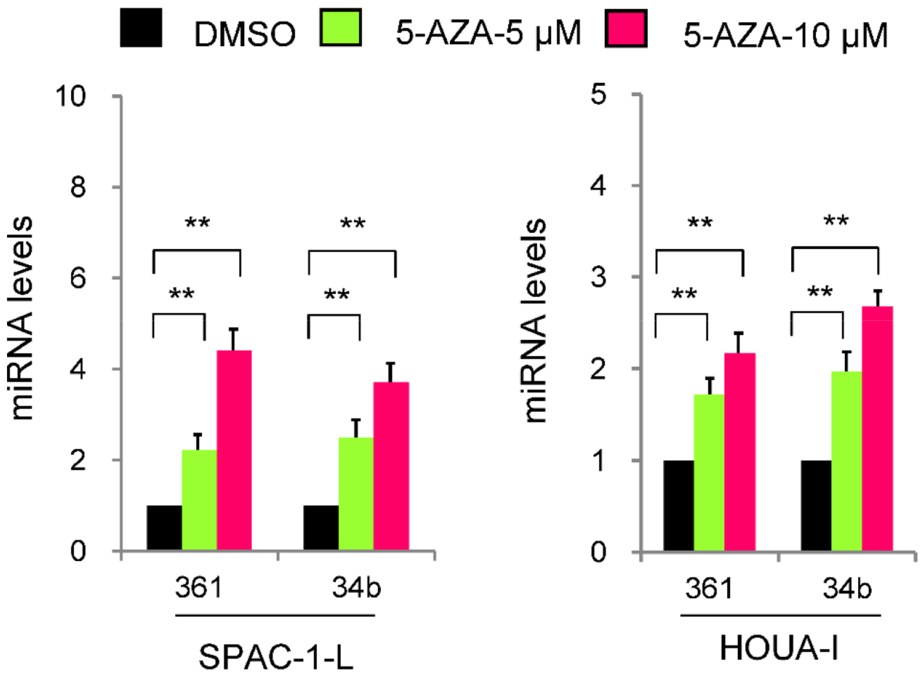

Supplementary Figure 10: 5-AZA induced miR-361 and miR-34b levels in EC cells. Expression of miR-361 and miR-34b in SPAC-1-L and HOUA-I cells after 5-AZA treatment (5 and 10 µM, 72 hours), as measured by qPCR. $^{**}P < 0.01$.
